# Same-day discharge vs. overnight stay following catheter ablation for atrial fibrillation: a comprehensive review and meta-analysis by the European Heart Rhythm Association Health Economics Committee

**DOI:** 10.1093/europace/euae200

**Published:** 2024-07-30

**Authors:** Maura M Zylla, Jacopo F Imberti, Francisco Leyva, Ruben Casado-Arroyo, Frieder Braunschweig, Helmut Pürerfellner, José L Merino, Giuseppe Boriani

**Affiliations:** Department of Cardiology, Heidelberg Center of Heart Rhythm Disorders, Medical University Hospital, Im Neuenheimer Feld 410, Heidelberg, Germany; Health Economics Committee of EHRA (European Heart Rhythm Association); Cardiology Division, Department of Biomedical, Metabolic and Neural Sciences, University of Modena and Reggio Emilia, Policlinico di Modena, Via del Pozzo 71, 41121 Modena, Italy; Clinical and Experimental Medicine PhD Program, University of Modena and Reggio Emilia, Modena, Italy; Health Economics Committee of EHRA (European Heart Rhythm Association); Aston Medical Research Institute, Aston Medical School, Aston University, Aston Triangle, B4 7ET Birmingham, UK; Health Economics Committee of EHRA (European Heart Rhythm Association); Department of Cardiology, H.U.B. Hôpital Erasme, Université Libre de Bruxelles, 1070 Brussels, Belgium; Health Economics Committee of EHRA (European Heart Rhythm Association); Department of Medicine, Solna, Karolinska Institutet; ME Cardiology, Karolinska University Hospital, Norrbacka S1:02, Eugeniavagen 27, 171 77 Stockholm, Sweden; Department of Cardiology, Public Hospital Elisabethinen, Academic Teaching Hospital, Ordensklinikum A-4020 Linz, Fadingerstraße 1, Austria; Arrhythmia-Robotic Electrophysiology Unit, La Paz University Hospital, IdiPAZ, Universidad Autonoma, Madrid, Spain; Health Economics Committee of EHRA (European Heart Rhythm Association); Cardiology Division, Department of Biomedical, Metabolic and Neural Sciences, University of Modena and Reggio Emilia, Policlinico di Modena, Via del Pozzo 71, 41121 Modena, Italy

**Keywords:** Atrial fibrillation, Catheter ablation, Complications, Mortality, Re-hospitalization, Same-day discharge

## Abstract

**Aims:**

Same-day discharge (SDD) after catheter ablation of atrial fibrillation (AF) may address the growing socio-economic health burden of the increasing demand for interventional AF therapies. This systematic review and meta-analysis analyses the current evidence on clinical outcomes in SDD after AF ablation compared with overnight stay (ONS).

**Methods and results:**

A systematic search of the PubMed database was performed. Pre-defined endpoints were complications at short-term (24–96 h) and 30-day post-discharge, re-hospitalization, and/or emergency room (ER) visits at 30-day post-discharge, and 30-day mortality. Twenty-four studies (154 716 patients) were included. Random-effects models were applied for meta-analyses of pooled endpoint prevalence in the SDD cohort and for comparison between SDD and ONS cohorts. Pooled estimates for complications after SDD were low both for short-term [2%; 95% confidence interval (CI): 1–5%; *I*^2^: 89%) and 30-day follow-up (2%; 95% CI: 1–4%; *I*^2^: 91%). There was no significant difference in complications rates between SDD and ONS [short-term: risk ratio (RR): 1.62; 95% CI: 0.52–5.01; *I*^2^: 37%; 30 days: RR: 0.65; 95% CI: 0.42–1.00; *I*^2^: 95%). Pooled rates of re-hospitalization/ER visits after SDD were 4% (95% CI: 1–10%; *I*^2^: 96%) with no statistically significant difference between SDD and ONS (RR: 0.86; 95% CI: 0.58–1.27; *I*^2^: 61%). Pooled 30-day mortality was low after SDD (0%; 95% CI: 0–1%; *I*^2^: 33%). All studies were subject to a relevant risk of bias, mainly due to study design.

**Conclusion:**

In this meta-analysis including a large contemporary cohort, SDD after AF ablation was associated with low prevalence of post-discharge complications, re-hospitalizations/ER visits and mortality, and a similar risk compared with ONS. Due to limited quality of current evidence, further prospective, randomized trials are needed to confirm safety of SDD and define patient- and procedure-related prerequisites for successful and safe SDD strategies.

## Graphical Abstract

**Graphical Abstract:**
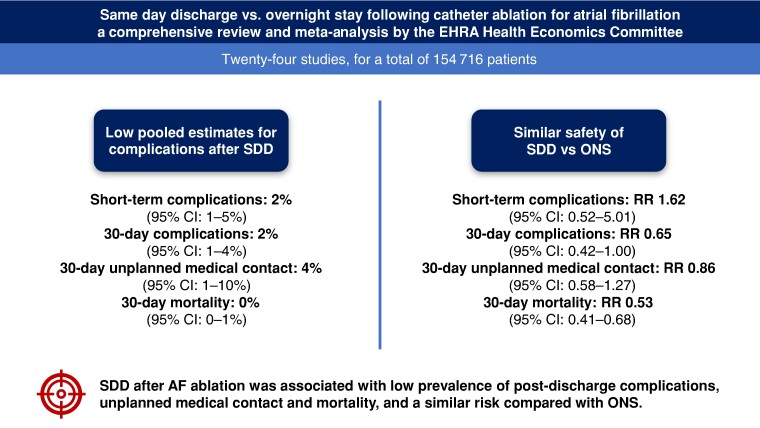

**What’s new?**

- This is the largest meta-analysis evaluating same-day discharge (SDD) vs. overnight stay (ONS) protocols after atrial fibrillation (AF) ablation so far, encompassing 24 studies (including two new randomized controlled trials) and 154 716 patients.
- By inclusion of additional recent evidence, short-term complication rates could be compared between SDD and ONS in addition to 30-day outcome.
- Short-term and 30-day complications associated with SDD after AF ablation are rare events and not elevated in comparison with ONS cohorts.
- Mortality after AF ablation is limited to single cases and not increased in SDD cohorts.
- Same-day discharge was not associated with an increase in post-discharge unplanned medical contacts or re-hospitalization.
- Due to the low quality of current evidence and heterogeneity regarding patient-related eligibility criteria and peri-SDD protocols, further large-scale prospective, randomized trials should be conducted in order to confirm the safety of SDD after AF ablation specific subgroups of patients.

## Introduction

Due to the increasing prevalence of atrial fibrillation (AF) in an ageing population, the demand for effective and safe therapeutic strategies is continuously growing. Catheter ablation of AF has been shown to provide superior efficacy with respect to rhythm control compared with pharmacological antiarrhythmic therapy.^1–3^ Additionally, there has been evidence of beneficial prognostic effects of catheter ablation in selected subgroups of patients.^4–6^

In light of the widespread implementation of AF ablation and increasing operator experience over the last decades, procedure durations and rates of severe procedural complications have substantially decreased in experienced centrws.^7^ Furthermore, novel technologies for AF ablation have reduced procedure times and can be performed under conscious sedation or minimal duration of intensified anaesthesia.^8–11^ Nevertheless, a post-procedural monitoring period of 12–24 h has been recommended in the guidelines of the American Heart Association to allow for the detection of potential complications, e.g. pericardial effusion or bleeding complications, and discharge practices are heterogeneous across centres.^12,13^ However, most procedure-related complications associated with AF ablation have been described to occur within the first 6 h after the ablation procedure.^14^ Due to the increasing case volume with indication for AF ablation coinciding with a limitation in structural resources (e.g. medical personnel and hospital beds), shortening of peri-procedural hospitalization and monitoring periods have been discussed.^13,15,16^ Apart from health economic advantages, shorter in-hospital stays may also improve patient satisfaction.^16^ However, many patients undergoing AF ablation are older or suffer from other co-morbidities that may make them more prone to peri-procedural complications as well as adverse effects of sedation or general anaesthesia.^17^

In analogy to other interventional therapies, same-day discharge (SDD) or shortening of in-hospital monitoring may enhance treatment capacity, provided that necessary peri-procedural safety is maintained. In this context, recent studies evaluated the feasibility and safety of SDD in comparison with at least one overnight stay (ONS) in AF ablation.

The aim of the present systematic review and meta-analysis is to describe the outcome in patients undergoing SDD protocols after catheter ablation for AF, based on the most recent evidence.^18–39^ The pooled estimates of complications, re-hospitalization/emergency room (ER) visits, and mortality after SDD are described and compared with ONS protocols.

## Methods

The present systematic review and meta-analysis was conducted in accordance with the Preferred Reporting Items for Systematic Reviews and Meta-Analysis (PRISMA) 2020 guidelines.^40^

### Eligibility criteria

Randomized controlled trials (RCTs) and prospective observational or retrospective studies analysing patients ≥ 18 years specifically undergoing AF ablation were included. Studies with a two-cohort design comparing SDD with ONS were used for the meta-analysis comparing pre-defined endpoints between these two discharge strategies. Studies investigating the outcome of a single SDD cohort without a comparator group were also eligible and used for descriptive analyses and meta-analyses of proportions. Studies reporting data on at least one of the pre-specified endpoints were included.

### Search strategy and study selection

We searched the PubMed database for studies fulfilling the eligibility criteria using the search string ‘atrial fibrillation’ AND (‘ablation’ OR ‘pulmonary vein isolation’) AND (‘same day discharge’ OR ‘day case’ OR ‘ambulatory’ OR ‘outpatient’). Regularly updated searches were performed during the preparation of the manuscript until 30 January 2024. The database was searched from inception, with no language restriction. Two authors (M.M.Z. and J.F.I.) independently screened all articles retrieved from the literature search based on title and abstracts using a standardized, web-based platform (Rayyan Systems Inc., Cambridge, MA, USA). Afterwards, full-text evaluation of pertinent citations was performed. Any discrepancies were resolved by collegiate discussion and consensus.

### Data collection and endpoints

Whenever available, data were extracted using a pre-specified electronic form and included study design, demographic and clinical baseline parameters, procedural characteristics, procedure-related complications, mortality, and successful SDD at discharge. Pre-defined endpoints for meta-analyses were (i) short-term complications (24–96 h), (ii) complications at 30-day post-discharge (leading to medical contact or with need for intervention), (iii) re-hospitalization and/or ER visits at 30-day post-discharge, and (iv) 30-day mortality. Complications were assessed according to the respective study-specific protocol. Intra-procedural or acute, in-hospital post-procedural complications were specifically distinguished from short-term complications after discharge as far as possible based on the data reported, as these were judged as rather associated with the procedure itself and not with the safety of the respective discharge strategy. The rate of successfully realized planned SDD strategy was evaluated in a descriptive fashion. In single cases of discrepancy between follow-up durations for endpoints assessment reported by the studies and pre-defined time points in this analysis, endpoint occurrence was derived from the description of timings, if available, or endpoints were categorized to the nearest corresponding time point if the difference was <3 days.

### Quality assessment and risk of bias

M.M.Z and J.F.I. performed quality assessment using the Newcastle–Ottawa Scale for non-randomized clinical trials and V.2 of the Cochrane ‘Risk of Bias’ tool (RoB2) for RCTs.^41,42^ The *Robvis* internet-based graphic generating platform was used to create the risk of bias plot with the results from RoB2.^43^

Studies with a score of ≤7 of the Newcastle–Ottawa Scale were categorized at significant risk of bias.

### Statistical analysis

In the SDD cohort, the prevalence of complications, ER admissions/re-hospitalization, and death was logit transformed. The pooled prevalence was computed using a random-effects method with an inverse variance approach. For direct comparison of SDD and ONS, the Mantel–Haenszel random-effects model was used to determine the pooled effect sizes for the outcomes of interest. Dichotomous outcomes were presented as risk ratio (RR) and the corresponding 95% confidence interval (CI). The outcomes were analysed on an intention-to-treat basis whenever possible. In order to measure heterogeneity, we calculated the inconsistency index (*I*^2^) and the tau (restricted maximum-likelihood method). Heterogeneity was defined as low if *I*^2^ was <25%, moderate if between 25% and 75%, and high if >75%.^44^

For each outcome, a sensitivity analysis was performed if *I*^2^ was >25% and the ‘leave-one-out’ approach was used (all studies were removed one at a time to assess their influence on pooled estimates and heterogeneity). Other potential sources of heterogeneity were explored through subgroups analyses including study type (retrospective vs. prospective studies and administrative data vs. non-administrative data) and SDD as a default approach vs. non-default.

Publication bias was assessed by visual inspection of funnel plots and Egger’s test when 10 or more studies were included. All statistical analyses were performed using R version 4.0.3 (The R Foundation, 2020), using the ‘meta’, ‘metafor’, and ‘dmetar’ packages.^45^

## Results

### Study selection and characteristics

**Figure 1:**
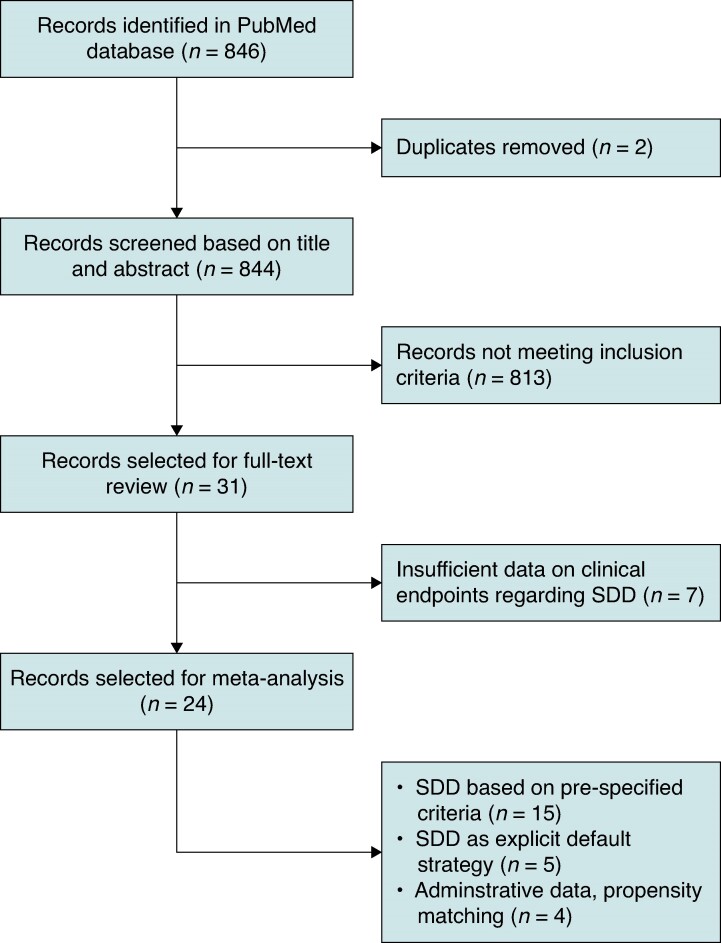
Study selection process (PRISMA flowchart). SDD, same-day discharge.

A total of 846 records were retrieved from the literature search. The time of publication ranged from 10/2010 to 11/2023. After the removal of duplicates and screening of titles and abstracts, 31 full-text articles were assessed for eligibility, of which 24 were included in the quantitative analysis (*Figure 1*). The majority of studies (*n* = 15) were retrospective, of which four analyses were based on administrative databases. Seven investigations were prospective observational studies. Additionally, two RCTs were identified. Fifteen studies were performed in the USA or Canada, seven in Europe, one in Argentina, and one across two continents (*Table 1*). Three studies analysing only outcome after SDD in a single cohort (one retrospective and two prospective) were included in the meta-analysis of proportions. Follow-up durations described in the selected studies ranged from 10 days to 6 months.

**Table 1:** Study characteristics

Study	Year	Study type/data source	Country	Sponsor	Total sample size (SDD/ONS), *n*
Haegeli *et al*.	2010	Retrospective, single centre	Canada	None	230 (205/0)
Ignacio *et al*.	2018	Prospective, single centre	Argentina	None	195 (58/137)
Bartoletti *et al*.	2019	Retrospective, single centre	UK	None	811 (143/642)
Opel *et al*.	2019	Prospective observational, two centres	UK	None	552 (276/276)
Creta *et al*.	2020	Retrospective, multicentre	UK	None	2628 (727/1901)
N Akula *et al*.	2020	Retrospective, single centre	USA	None	571 (426/145)
Reddy *et al*.	2020	Retrospective, single centre	UK	None	452 (128/323)
Deyell *et al*.	2020	Retrospective, two centres	Canada	None	3054 (2418/636)
Brown *et al*.	2021	Retrospective, two centres	USA	None	409 (210/199)
Field *et al*. HR	2021	Retrospective, administrative data, propensity matching	USA	Johnson and Johnson	6247 (1610/4637)
Field *et al*. JCE	2021	Retrospective, administrative data, propensity matching	USA	Johnson and Johnson	6600 (1660/4940)
Kowalski *et al*.	2021	Retrospective, propensity matching, multicentre	USA	None	2374 (1194/1180)
Rajendra *et al*.	2021	Prospective, propensity matching, multicentre	USA	Biosense Webster	82 (41/41)
He *et al*.	2021	Retrospective, multicentre	UK/Qatar	None	967 (414/553)
Sahashi *et al*.	2022	Retrospective, administrative data, propensity matching	USA	None	1751 (440/1311)
Castro-Urda *et al*.	2023	Randomized controlled, single centre	Spain	None	100 (50/50)
Rajendra *et al*.	2023	Prospective, propensity matching, multicentre	USA	None	2332 (1982/350)
Asbeutah *et al*.	2023	Retrospective, two centres	USA	None	225 (91/134)
Obeid *et al*.	2023	Retrospective, administrative data	USA	None	122 289 (101 162/21 127)
Sangrigoli *et al*.	2023	Randomized controlled, multicentre	USA	Medtronic	45 (22/23)
Eldadah *et al*.	2023	Prospective, multicentre	USA	Cardiva Medical, Inc (now part of Haemonetics Corporation)	354 (323/31)
Deyell *et al*.	2023	Retrospective, two centres	Canada	None	427 (381/40)
Jimenez-Candil *et al*.	2023	Prospective, single centre	Spain	Biomedical Research Institute of Salamanca	617 (439/0)
Honarbakhsh *et al*.	2023	Prospective, multicentre	UK	None	1688 (1641/0)

ONS, overnight stay; SDD, same-day discharge.

### Patient population

A total of 116 041 patients underwent AF ablation with SDD and 38 676 with ONS across 24 studies. Mean age ranged between 56 and 67 years in the SDD cohort and between 59 and 68 years in the ONS cohort, in studies comparing these two strategies. In the majority of studies, male sex was predominant in both SDD and ONS cohorts (*Table 2*). Data on AF type were available for 16 studies evaluating SDD patients and 10 studies evaluating ONS patients. Paroxysmal AF was the most common AF type for both patient populations (*Table 2*). Other available baseline parameters, e.g. CHA_2_DS_2_-VASc-score, body mass index, and left ventricular ejection fraction, were reported in only few studies and corresponded to characteristics of typical AF ablation cohorts (*Table 2*).

**Table 2:** Patient population characteristics

Study	Age SDD^a^ (years)	Age ONS^a^ (years)	Male SDD *n* (%)	Male ONS *n* (%)	Paroxysmal AF SDD *n* (%)	Paroxysmal AF ONS *n* (%)	CHA_2_DS_2_-VASc SDD^a^	CHA_2_DS_2_-VASc ONS^a^	BMI SDD^a^	BMI ONS^a^	LVEF SDD^a^ (%)	LVEF ONS^a^ (%)
Haegeli *et al*., 2010	56	n/a	152 (74)	n/a	171 (83)	n/a	n/a	n/a	n/a	n/a	59	n/a
Ignacio *et al*., 2018	57	62	49 (85)	103 (75)	46 (79)	108 (79)	n/a	n/a	28	27	60	60
Bartoletti *et al*., 2019	59	59	98 (69)	437 (68)	108 (76)	n/a	2.0	1.0	30	29	n/a	n/a
Opel *et al*., 2019	60	n/a	168 (61)	n/a	218 (79)	n/a	1.8	n/a	n/a	n/a	n/a	n/a
Creta *et al*., 2020	61	63	499 (69)	1142 (60)	422 (58)	928 (49)	n/a	n/a	n/a	n/a	n/a	n/a
N Akula *et al*., 2020	62	60	298 (70)	86 (59)	247 (58)	103 (71)	n/a	n/a	n/a	n/a	n/a	n/a
Reddy *et al*., 2020	60	n/a	90 (70)	236 (73)	105 (81)	210 (65)	n/a	n/a	n/a	n/a	n/a	n/a
Deyell *et al*., 2020	60	n/a	2224 (73)	n/a	1907 (62)	n/a	n/a	n/a	n/a	n/a	n/a	n/a
Brown *et al*., 2021	65	63	143 (68)	134 (67)	128 (61)	97 (49)	n/a	n/a	29	31	n/a	n/a
Field *et al*. HR, 2021	n/a	n/a	1247 (78)	3604 (78)	n/a	n/a	1.6	1.6	n/a	n/a	n/a	n/a
Field *et al*. JCE, 2021	n/a	n/a	1101 (66)	3264 (66)	n/a	n/a	3.0	3.0	n/a	n/a	n/a	n/a
Kowalski *et al*., 2021	64	66	825 (69)	93 (67)	n/a	n/a	1.4	2.2	30	31	56	55
Rajendra *et al*., 2021	59	60	25 (61)	25 (61)	41 (100)	41 (100)	1.6	1.6	30	30	n/a	n/a
He *et al*., 2021	63	60	187 (45)	208 (43)	267 (65)	353 (64)	n/a	n/a	n/a	n/a	n/a	n/a
Sahashi *et al*., 2022	67	66	252 (57)	712 (54)	n/a	n/a	n/a	n/a	n/a	n/a	n/a	n/a
Castro-Urda *et al*., 2023	65	63	32 (64)	37 (74)	29 (58)	28 (56)	n/a	n/a	27	27	56	57
Rajendra *et al*. 2023	65	68	1195 (60)	193 (55)	n/a	n/a	2.8	3.1	31	31	55	51
Asbeutah *et al*., 2023	n/a	n/a	n/a	n/a	n/a	n/a	n/a	n/a	n/a	n/a	n/a	n/a
Obeid *et al*., 2023	n/a	n/a	n/a	n/a	n/a	n/a	n/a	n/a	n/a	n/a	n/a	n/a
Sangrigoli *et al*., 2023	59	62	15 (68)	17 (74)	22 (100)	23 (100)	n/a	n/a	n/a	n/a	n/a	n/a
Eldadah *et al*., 2023	65	66	231 (72)	22 (71)	138 (43)	13 (42)	2.3	3.1	30	31	n/a	n/a
Deyell *et al*., 2023	n/a	n/a	n/a	n/a	n/a	n/a	n/a	n/a	n/a	n/a	n/a	n/a
Jimenez-Candil *et al*., 2023	n/a	n/a	439 (71)	n/a	316 (51)	n/a	n/a	n/a	n/a	n/a	n/a	n/a
Honarbakhsh *et al*., 2023	62	n/a	1119 (66)	n/a	1158 (69)	n/a	n/a	n/a	n/a	n/a	n/a	n/a

AF, atrial fibrillation; BMI, body mass index; LVEF, left ventricular ejection fraction; n/a, not available; ONS, overnight stay; SDD, same-day discharge.

^a^Mean or median.

### Procedural characteristics

The majority of studies investigated outcome after both radiofrequency (RF) and cryoballoon (CB) ablation of AF (*n* = 13), four studies each reported data from exclusively RF ablation or CB ablation, and in three studies, information on ablation technique used was missing (*Table 3*).

**Table 3:** Procedural characteristics

Study	Ablation technique	Type of anaesthesia	Peri-procedural/intra-procedural anticoagulation	Pre-procedural criteria for SDD	Post-procedural criteria for SDD	SDD successful as planned n/total (%)
Haegeli *et al*., 2010	RF	Conscious sedation	VKA discontinued 3 days before the procedure, INR target <2.0, and anticoagulation restarted on the same day of the procedure/ACT 250–300s	Absence of significant structural heart disease	n/a	205/230 (89.1)
Ignacio *et al*., 2018	Cryo/RF	General anaesthesia	DOAC stopped 24 h before the procedure; for VKA INR target 2–2.5/ACT 350–450s	No specific exclusion criteria for SDD	n/a	n/a
Bartoletti *et al*., 2019	Cryo/RF	General anaesthesia/conscious sedation	DOAC morning dose omitted and restarted the evening post procedure; uninterrupted VKA (INR ≤ 3.5)/ACT ≥ 300 s and protamine reversal	n/a	(i) No significant concerns, (ii) absence of LV dysfunction or a recent history of uncontrolled HF, (iii) patient choice, and (iv) procedure end before 14:00 h	n/a
Opel *et al*., 2019	Cryo	General anaesthesia/conscious sedation	Uninterrupted OAC/standardized i.v. heparin dose	First time ablation	Nurse-led, after 4-h recovery in absence of complications at echocardiography	272/276 (98.6)
Creta *et al*., 2020	Cryo/RF	Mostly conscious sedation	Uninterrupted OAC/ACT 300–350 s	Excluded pts based on social, geographic and clinical factors, the very elderly or other major co-morbidities considered to preclude safe SDD	(i) Operator decision and (ii) nurse-led, after 4-h recovery in absence of complications	n/a
N. Akula *et al*., 2020	RF	General anaesthesia/conscious sedation	Not standardized	n/a	After 6-h monitoring, SDD cancelled if patient had any ablation related complications, non-ablation related medical care or due to patient specific/social reasons	376/426 (88.3)
Reddy *et al*., 2020	Cryo/RF	General anaesthesia/conscious sedation	One omitted DOAC dose pre-procedure; uninterrupted VKA/ACT > 300 s and protamine reversal	(i) Social support post-discharge and (ii) subjective selection based on frailty, co-morbidities, distance, and patient wishes	(i) Acceptable groin haemostasis, (ii) clinically stable and (iii) suitable caregiver present on discharge	128/129 (99.2)
Deyell *et al*., 2020	Cryo/RF	Mostly general anaesthesia	Not standardized	n/a	(i) Treating physician and nursing team discretion and (ii) stable vital signs, no access bleeding, ambulation tolerated, support person at home	2418/3054 (79.2)
Brown *et al*., 2021	Cryo/RF	GA/conscious sedation	n/a	n/a	2–4 h of bed rest, clinical exam, and groin check	182/257 (70.1)
Field *et al*. Heart Rhythm 02, 2021	n/a	n/a	n/a	n/a	n/a	n/a
Field *et al*. JCE, 2021	n/a	n/a	n/a	n/a	n/a	n/a
Kowalski *et al*., 2021	Cryo	General anaesthesia	Not standardized/ACT ≥ 300 s and protamine reversal	n/a	Centre-specific, in short: absence of complications, circulatory and respiratory stability after 6h monitoring and arrangements for ‘secure-to-home’ transportation	n/a
Rajendra *et al*., 2021	RF	General anaesthesia	Uninterrupted OAC/n/a	(i) Stable anticoagulation, (ii) absence of bleeding history, (iii) no systolic heart failure, (iv) no history of pulmonary disease, (v) no interventional procedures within 60 days, (vi) body mass index < 35 kg/m^2^ (recommended), (vii) acceptable CHA2DS2-VASc score (‘typically’ ≤ 3), (viii) patient proximity to hospital and willingness to stay at a nearby hotel if needed, considered, (ix) ablation prior 1:00 PM	(i) No complications, (ii) operator confirmation to SDD, (iii) purse string suture removed, (iv) stable haemodynamics, (v) no groin or respiratory complications, (vi) able to tolerate liquids/food, and (vii) able to ambulate	41/44 (93.2)
He *et al*., 2021	Cryo/RF	Mostly conscious sedation	For once-daily and twice-daily DOACs last dose 20- and 10-h pre-procedure; for VKA, INR 2–3.5 on procedure’s day/n/a	No specific exclusion criteria for SDD	n/a	328/414 (79.5%)
Sahashi *et al*., 2022	n/a	n/a	n/a	n/a	n/a	n/a
Castro-Urda *et al*., 2022	Cryo/RF	Conscious sedation	Uninterrupted OAC/ACT > 300 s	n/a	Randomization to vascular closure system utilization and SDD vs. traditional closure and ONS	42/50 (84%)
Rajendra *et al*. 2023	RF	General anaesthesia	Uninterrupted OAC/n/a	(i) Stable anticoagulation, (ii) absence of bleeding history, (iii) LVEF > 40%, (iv) no history of pulmonary disease, (v) no interventional procedures within 60 days, and (vi) BMI < 35 kg/m^2^	(i) No complications, (ii) stable haemodynamics, (iii) no groin or respiratory complications, (iv) able to ambulate and tolerate liquids/food, (v) purse-string suture removed if applicable, and (vi) appropriate social support	1707/1982 (86.1%)
Asbeutah *et al*., 2023	Cryo/RF	n/a	n/a	No specific exclusion criteria for SDD (subgroup from COVID-19 pandemic compared with pre-pandemic subgroup)	n/a	n/a
Obeid *et al*., 2023	Cryo/RF	n/a	n/a	n/a	n/a	n/a
Sangrigoli *et al*., 2023	Cryo	General anaesthesia	n/a/ACT > 350s and reversal	(i) ≥18 years old, (ii) LVEF > 40%, (iii) GFR > 50, and (iv) CHA_2_DS_2_VASC ≤ 3,	(i) Uncomplicated procedure with <4 h of GA and (ii) no complications or incidence of early AF during 6-h post-ablation observation period	22/22 (100%)
Eldadah *et al*., 2023	Cryo/RF	n/a	n/a	(i) ≥18 years old, (ii) no active systemic or cutaneous infection near the access site(s), history of venous thromboembolic events, bleeding diathesis, coagulopathy, hypercoagulability, or platelet count <100 000 cells/mm^3^, femoral catheterization in any study limb in the previous 30 days, planned procedure(s) or concomitant condition(s) that may delay ambulation or hospital discharge, use of LMWH within 8 h, and procedural details that may delay ambulation or hospital discharge	At operator discretion if the following criteria were met: formal discharge evaluation, successful ambulation without bleeding from the access site, ability to void, no clinically significant ECG findings, and presence of a responsible adult for 24 h	323/354 (99.1%)
Deyell *et al*., 2023	Cryo/RF	General anaesthesia	n/a	No specific exclusion criteria for SDD	SDD as default strategy for all patients, unless a pre-planned ONS was requested by the treating physician in cases of medical complexity or lack of social supports	381/421 (90.5)
Jimenez-Candil *et al*., 2023	Cryo/RF	Conscious sedation	Morning OAC dose omitted and restarted the afternoon of the procedure/ACT > 300 s	n/a	(i) At operator discretion, (ii) no intra- post-procedural complications, (iii) 3-h bed rest, mobilization, figure-of-eight’ suture removed, and clinical check-up, including echocardiography, and (iv) usual time of procedure before 3 PM	585/617 (94.8)
Honarbakhsh *et al*., 2023	Cryo	General anaesthesia/conscious sedation	Uninterrupted OAC/ACT > 300 s	No specific exclusion criteria for SDD	No specific exclusion criteria for SDD, and the inability to discharge the patient post-procedure was dependent on the procedural outcome and recovery post-procedure	1641/1688 (97.2)

ACT, activated clotting time; BMI, body mass index; Cryo, cryoballoon ablation; DOAC, direct oral anticoagulant; GFR, glomerular filtration rate; n/a, not available; LA, left atrial; LV, left ventricular, LVEF, left ventricular ejection fraction, OAC, oral anticoagulants; ONS, overnight stay; PVI, pulmonary vein isolation; RF, radiofrequency ablation; SDD, same-day discharge; TEE, trans-oesophageal echocardiography; VKA, vitamin K antagonist.

Peri-procedural sedation was performed using general anaesthesia in six studies, conscious sedation in another three studies, and variable protocols using either general anaesthesia or conscious sedation were reported by nine studies. In six studies, information on peri-procedural sedation was not available (*Table 3*).

Fourteen studies provided information on peri- or intra-procedural anticoagulation. Ten studies specified intra-procedural application of unfractionated heparin with monitoring of activated clotting time, and three studies reported routinely performed protamine reversal. Uninterrupted or minimally interrupted use of oral anticoagulation was reported by nine studies. Two studies described peri-procedural anticoagulation as guideline directed or performed as by the discretion of the operator. In the remaining studies, information on peri-procedural anticoagulation regimen was not specified (*Table 3*).

### Criteria for same days discharge and success of planned discharge strategy

In studies reporting protocols for SDD, pre-specified eligibility criteria for this discharge strategy varied considerably (*Table 3*). Most common criteria applied were full recovery and clinical stability after a post-procedural monitoring period of 4–6 h, as well as the absence of procedural complications. Other criteria reported were the absence of significant co-morbidities potentially predisposing for procedural complications, stable anticoagulation without signs of bleeding or history of bleeding events, place of residence in proximity to the ablation centre, early finish of ablation procedure, or presence of competent support at home (*Table 3*). Five studies report SDD as a default strategy without previously defined patient-specific criteria. However, SDD still depended on post-procedural outcome or was subject to operator discretion (*Table 3*). Successful realization of planned discharge strategy, if reported, was achieved in the majority of SDD cases, ranging from 70.1% to 100% (*Table 3*).

### Short-term complications post-discharge

The pooled prevalence of short-term complications after SDD was 2% (95% CI: 1–5%), reported in 4728 patients across 12 studies with high heterogeneity (*I*^2^: 89%; *τ*^2^: 1.48; 95% CI: 0.60–7.43) (see Supplementary material online, *Figure S1*). In sensitivity analyses, the study by Brown *et al*. was the most relevant contributor to the heterogeneity, but we did not observe significant influence of single studies on the pooled estimates (see Supplementary material online, *Figure S2*). Subgroup analyses showed a similar prevalence of short-term complications among retrospective and prospective studies (1%; 95% CI: 0–7%; *I*^2^: 91% vs. 2%; 95% CI: 1–5%; *I*^2^: 79%) (see Supplementary material online, *Figure S3*).

**Figure 2:**
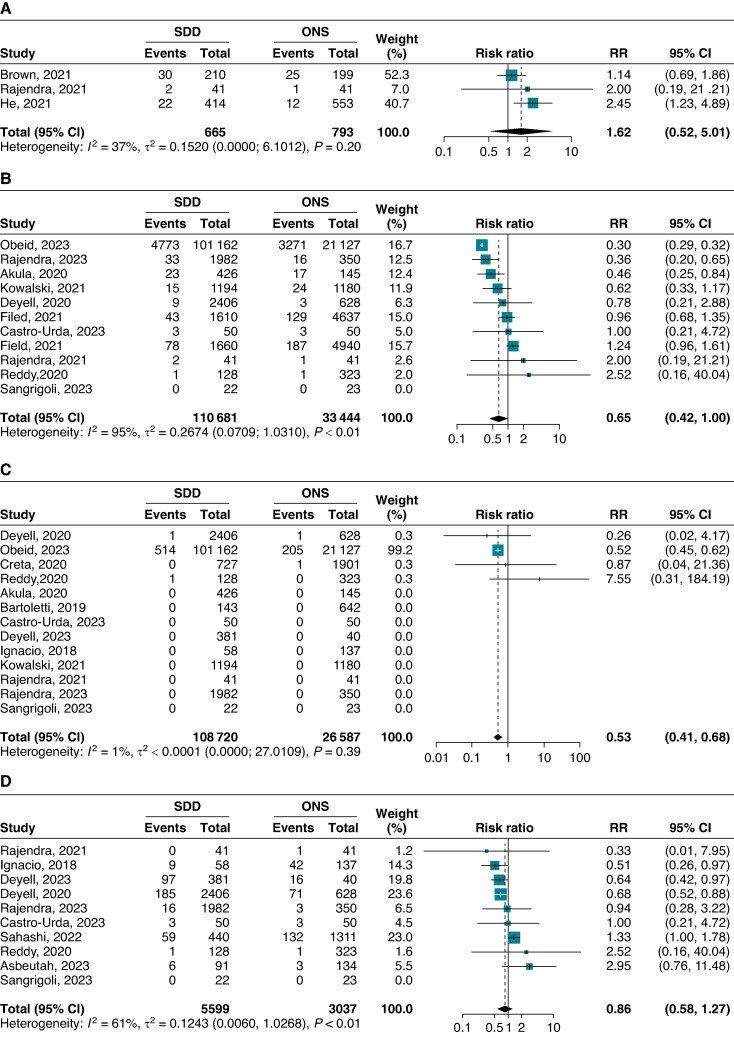
Forest plots showing the comparative safety of same-day discharge strategy vs. overnight stay after catheter ablation of atrial fibrillation. (*A*) Risk of short-term complications. (*B*) Risk of complications at 30 days. (*C*) Risk of death at 30 days. (*D*) Risk of unplanned medical contact at 30 days. CI, confidence ratio; ONS, overnight stay; RR, risk ratio; SDD, same-day discharge.

Three studies provided comparative data on short-term complications in ONS or SDD in a total of 1458 patients. The pooled risk of short-term complications was not statistically different between the two discharge strategies (RR: 1.62; 95% CI: 0.52–5.01), with moderate heterogeneity (*I*^2^: 37%; *τ*^2^: 0.15; 95% CI: 0.00–6.10) (*Figure 2A*). Reported complications at 24 h included vascular and bleeding complications, pericarditic chest pain, pericardial effusion, AF recurrence, nausea, infection, stroke, and pulmonary oedema.

### Complications at 30-day post-discharge

Sixteen studies, including a total of 113 698 patients, reported complications at 30 days after SDD. The pooled prevalence was 2% (95% CI: 1–4%), with high heterogeneity (*I*^2^: 91%; *τ*^2^: 0.86; 95% CI: 0.39–2.60) (see Supplementary material online, *Figure S4*). No single study significantly influenced the pooled estimates or heterogeneity (see Supplementary material online, *Figure S5*). Subgroup analyses of retrospective and prospective studies revealed no significant influence of study design regarding heterogeneity or pooled estimates (see Supplementary material online, *Figure S6*).

In comparison with ONS, SDD showed not statistically significant difference with respect to complications at 30-day post-discharge (RR: 0.65; 95% CI: 0.42–1.00), with high heterogeneity (*I*^2^: 95%; *τ*^2^: 0.27; 95% CI: 0.07–1.03) across 11 studies in 144 125 patients (*Figure 2B*). In sensitivity analysis, the study by Obeid *et al*. was the most relevant contributor to heterogeneity. However, omitting this study resulted in no statistically significant difference between ONS and SDD strategies regarding this endpoint with a reduction in heterogeneity between studies (see Supplementary material online, *Figure S7*). In subgroup analyses, lower heterogeneity was found among prospective studies as compared with retrospective studies (*I*^2^: 34% vs. *I*^2^: 96%), with similar pooled RRs (see Supplementary material online, *Figure S8A*). Similar results were observed among administrative studies (see Supplementary material online, *Figure S8B*).

Respiratory, cerebrovascular, peripheral vascular and pericardial complications, phrenic nerve damage, AF recurrence, myocardial infarction, and sepsis were reported as complications at 30-day post-discharge.

### Mortality at 30-day post-discharge

Fifteen studies (109 226 patients) reported mortality in the context of SDD after AF ablation. In 12 studies, no death was recorded at 30 days after SDD; only three studies reported cases of post-procedural death. Of these three studies, one retrospective analysis of early mortality offered no information on individual causes of death. Each of the other two studies described one case of post-procedural death: one patient died of a stroke at 24-day post-discharge; the other one died due to an atrio-oesophageal fistula 3 weeks after discharge. Pooled mortality rates were low (0%; 95% CI: 0–1%; *I*^2^: 33%; *τ*^2^: 0.48; 95% CI: 0.00–3.05) (see Supplementary material online, *Figure S9*). The study by Deyell *et al*. was the most significant contributor to heterogeneity for mortality, with no significant effect of single studies on the pooled estimates (see Supplementary material online, *Figure S10*). The study design was not a potential source of heterogeneity and did not result in significant changes in pooled estimates for the endpoint investigated (see Supplementary material online, *Figures S11*).

Mortality was reported in 13 studies comparing SDD vs. ONS (135 307 patients). Of note, no deaths were reported in 9/13 (69%) studies. Same-day discharge was associated with a lower pooled risk of mortality at 30-day post-discharge as compared with ONS (RR: 0.53; 95% CI: 0.41–0.68; *I*^2^: 1%; *τ*^2^: <0.01; 95% CI: 0.00–27.01) (*Figure 2C*). However, these results were driven mainly by one retrospective analysis of administrative data with limited information on causes of post-procedural mortality (see Supplementary material online, *Figures S12* and *S13*). This study also reported the presence of co-morbidities and procedural complications as predictors for early mortality after discharge. In ONS control groups, two causes of post-procedural death were described: one patient died of unclear cause one day after discharge; the other patient died of atrio-oesophageal fistula.

### Unplanned medical contact at 30-day post-discharge

Re-hospitalization rates and ER visits after SDD were recorded in 12 studies (6018 patients) with high heterogeneity (*I*^2^: 96%; *τ*^2^: 2.00; 95% CI: 0.77–6.62) and amounted to 4% (95% CI: 1–10%) in the pooled analysis (see Supplementary material online, *Figure S14*). Reasons for unplanned medical contact included arrhythmia recurrence, pericarditis or chest pain, vascular or bleeding complications, bradycardia, or respiratory complications. No single study significantly influenced the pooled estimates or heterogeneity for re-hospitalizations or ER visits (see Supplementary material online, *Figure S15*), nor did the study design (see Supplementary material online, *Figure S16*). Compared with ONS, SDD was not associated with an increase in re-hospitalization and ER visits with moderate heterogeneity between 10 studies (8636 patients) comparing these two discharge strategies (RR: 0.86; 95% CI: 0.58–1.27; *I*^2^: 61%; *τ*^2^: 0.12; 95% CI: 0.01–1.03) (*Figure 2D*). The study by Sahashi *et al*. and retrospective studies significantly contributed to the heterogeneity observed (see Supplementary material online, *Figures S17* and *S18*). The overall results of the main analysis did not substantially change when SDD was used as a default strategy vs. non-default strategy (see Supplementary material online, *Figure S19*).

### Bias assessment

Visual inspection of funnel plots and Egger’s test showed potentially missing studies for 30-day complications, in both the bottom right and bottom left sides (see Supplementary material online, *Figures S20* and *S21*). Therefore, pooled estimates are unlikely to change substantially with the addition of potential further studies. Significant publication bias was not observed for other outcomes (see Supplementary material online, *Figures S22* and *S23*).

All studies were considered at significant risk of bias, mostly due to the study design (non-randomized observational studies; 15 of 24 following a retrospective design) (see Supplementary material online, *Table S1*). Some concerns were observed also with regard to the two RCTs (see Supplementary material online, *Figure S24*).

The PRISMA checklist is reported in Supplementary material online, *Table S2*.

## Discussion

The increasing demand for catheter ablation of AF is associated with organizational, structural, and economic challenges. A shortening of peri-procedural hospital stay in selected patients may contribute to economizing capacities at ablation centres in order to support optimized patient care. This meta-analysis of current evidence including 24 studies and 154 716 patients of whom 116 041 underwent SDD shows a low risk for post-procedural complications, re-hospitalization/ER visits, and mortality associated with SDD after AF ablation. As lately several new studies have been added to the body of evidence on this clinically important topic, this meta-analysis includes 10 more studies compared with previous meta-analyses and, thus, encompasses the largest patient cohort analysed so far.^15,46^

Importantly, most of the evidence on SDD after AF ablation is still based on retrospective or observational studies. Only two RCTs could be identified, which, however, had not been published at the time when previous meta-analyses were conducted.^18,22^ The overall limited quality of evidence and relevant risk of bias has to be considered in the interpretation of the results. However, many large studies strove to correct for baseline differences, whenever present, between patient cohorts undergoing SDD or ONS.^21,29,30,36,37,39^ Additionally, considerable heterogeneity regarding SDD protocols, if reported, and potential bias whenever the discharge strategy was chosen at the discretion of the operator have to be taken into account. Furthermore, only few of studies reported SDD as a default strategy without any pre-defined conditions regarding patient-specific or peri-procedural SDD criteria. The latter varied considerably across the different studies. Nevertheless, the overall results remained consistent over multiple subgroup analyses, including administrative vs. non-administrative studies and studies in which SDD was a default strategy for ‘all-comers’ cohorts vs. those studies with pre-defined criteria for SDD selection. This corroborates the overall validity of the results.

In accordance to previous analyses, SDD was not associated with increased post-procedural risk for serious complications. Previous studies showed that the majority of peri-procedural complications in AF ablation occur before 6 h after the procedure.^14^ In accordance with these results, studies in this meta-analysis report no complications that could have been prevented by choosing an ONS discharge strategy over SDD.

Importantly, the seriousness of non-fatal complications reported and contributing to this endpoint varied across studies. We listed and considered all complications reported as they were judged as clinically relevant in the context of the respective studies. Thus, complications with no influence on the discharge strategy, e.g. minor bleeding complications, may have also contributed to the comparably high rate of 2% short-term complications in SDD cohorts.

Catheter ablation for AF is primarily set out to improve symptoms and quality of life, and prognostic benefits have not yet been shown for the broad spectrum of AF patients.^47^ Accordingly, preventing fatal complications is of utmost importance in the context of AF ablation. Importantly, fatal post-procedural events were limited to single cases in studies with follow-up on the cause of death and were not elevated in comparison with ONS cohorts. A trend towards lower mortality in SDD in comparison with ONS was mainly driven by one study with incomplete follow-up on the cause of death. Therefore, bias due to mortality related to co-morbidities in the ONS cohort rather than due to post-procedural complications is likely. Atrio-oesophageal fistula is one rare complication of AF ablation associated with high mortality and has been reported as one of the causes of death in the studies included. However, the evolvement of atrio-oesophageal fistula usually takes place over several weeks, and, thus, rather the procedure itself rather than the acute post-procedural monitoring period plays a role in preventing this serious complication.

Many implemented SDD programmes described in the analysed set of studies define a comprehensive list of criteria regarding eligible patients and procedural characteristics in order to qualify for SDD. Therefore, the results may not apply to all AF patients undergoing catheter ablation, in particular, if relevant co-morbidities are present or if an adequate duration of post-procedural monitoring cannot be provided, e.g. due to late procedure timing. The optimal protocol for patient screening or monitoring for SDD eligibility cannot be identified based on this analysis, not least due to the high heterogeneity of protocols reported by the studies. However, the presence of relevant co-morbidities predisposing for procedural complications, age, peri-procedural general anaesthesia or prolonged sedation, late timing of the procedure itself, and, potentially, also lack of social network to ensure adequate support and care after discharge may constitute factors that hamper SDD.^48^ Additionally, operator experience and structural and personal facilities at the ablation centre for post-procedural monitoring, pre-discharge clinical assessment, and patient counselling are important contributors for successful SDD. Interestingly, SDD was found safe also in the subgroup of studies in which SDD was applied as a default strategy. Even though some studies used general anaesthesia as part of the peri-procedural sedation protocol, also deep sedation administered by trained electrophysiologists staff during left atrial procedures has been reported as feasible and safe, and need for escalation to endotracheal intubation was low.^49,50^ Deep sedation may also be a valid sedation strategy for novel ablation technologies, including pulsed field ablation (PFA).^51^ Apart from patient-related characteristics, non-clinical logistical issues have been reported to constitute a major barrier for SDD.^52^ The use of single-shot devices or modern technologies like PFA may shorten duration of procedure and sedation, as well as operational complexity and perhaps costs, once well established in the market. This may on the other hand additionally support SDD.^53^ Of note, PFA SDD protocols have to be described yet, including details on each single technique used. Additionally, new technologies and particularly PFA have been associated with a relevant decrease in specific serious complications, like phrenic nerve palsy or atrio-oesophageal fistula.^53^ However, complications relating to vascular access or trans-septal puncture may still rather depend on operator experience and centre-specific protocols, e.g. the use of ultrasound guidance. Unfortunately, the underlying data of the present meta-analysis did not allow for a comparative analysis between single-shot and RF ablation strategies. Of note, the study by Deyell *et al*.^24^ directly compared SDD between 339 patients undergoing RF ablation as compared with 82 undergoing CB ablation. A similar proportion of patients achieved SDD (89.8 vs. 95.1%). Re-admission or ER visits within 30 days were not different between the two groups (26.2% vs. 29.2%; *P* = 0.63). These data have to be further evaluated in future large-scale prospective studies.

Atrial fibrillation constitutes a significant health burden in our society, and the demand for interventional AF therapies is rising.^47^ Therefore, an increasing socio-economic burden, as well as the demand for structural and personal resources related to AF ablation, can be expected. Studies comparing the economic characteristics of SDD and ONS strategies show substantial cost savings associated with SDD.^23,31,36,52^ The cost reduction achievable with saving hospital resources in SDD has been calculated to amount to up to 63%.^23^ Depending on the respective reimbursement system, the use of vascular closure devices in SDD protocols may lead to additional costs regarding implant material while savings can be achieved in facility, pharmacy, and other supplies and services.^54^ Nevertheless, most studies included in this meta-analysis report the safety of SDD with conventional sutures for vascular access site management. In the context of SDD, post-discharge unplanned medical contacts may also contribute to the economic burden associated with AF ablation. Deyell *et al*. report that health care utilization remains high both in SDD and ONS, particularly due to arrhythmia recurrence and post-procedural chest pain or pericarditis. However, rates of ER visits did not differ between SDD and ONS protocols in their study. Importantly, also our meta-analysis showed no increase in post-procedural medical contacts via emergency services or re-hospitalization in SDD compared with ONS.

Apart from potential structural and economic advantages, SDD after catheter ablation of AF has been shown to increase patient satisfaction.^55,56^ In a recent survey, 50% of eligible patients at a high-volume ablation centre would have favoured an SDD strategy after AF ablation.^56^ Major concerns, if present, were uncertainties regarding potential post-procedural complaints or recognition of post-discharge complications. This is in line with concerns reported by physicians, which also state the fear of increased re-hospitalization rates as one factor precluding the implementation of SDD protocols.^16^ Even though the data from this meta-analysis cannot confirm this notion, patients’ and physicians’ concerns additionally highlight the need for optimized processes and clinical assessment prior to discharge and, ideally, a concept of post-discharge contact, e.g. by nurse-led telephone contacts shortly after discharge as described in some studies included in this meta-analyses.^21,23,31,32^

Even though current evidence suggests that SDD constitutes a safe and feasible concept, SDD is implemented in only a minority of European centres.^13,16^ According to recent European surveys, national reimbursement practices partially explain favouring ONS, whereas bed availability has substantial influence on planning of elective procedures.^13,16^ Consequently, more robust evidence on patient selection, structural prerequisites, and, most importantly, safety of SDD is needed in order to evaluate its feasibility on a broad basis. Future randomized controlled studies to confirm the benefit of SDD after AF should focus on including patients representing a ‘real-world’ cohort. This is of particular importance as patients referred for AF ablation now and in the coming years are becoming older and suffer from more cardiac and non-cardiac co-morbidities, as the technologies are becoming safer and evidence for beneficial effects of AF ablation in specific high-risk cohorts, e.g. advanced heart failure, accumulates.^5^ Therefore, reduction of exclusion criteria regarding age and co-morbidities would strengthen the transferability of the results into clinical practice. Only if the barrier for SDD is lowered in a truly representative AF patient cohort, a relevant increase in SDD rates can be achieved, providing that reimbursement and structural prerequisites support this strategy. As the endpoints of serious complications and mortality are rare events after SDD, a RCT comparing SDD and ONS should rather target a combined endpoint including both clinical and patient-reported outcomes (e.g. quality of life and patient satisfaction) and resource utilization. This approach would have the benefit of allowing a smaller sample size and also test the fundamental assumption that the two strategies should be equally safe and not differ in terms of readmission rates. Accordingly, the results presented in this meta-analysis of recent evidence can be a basis for the conceptualization of such future scientific endeavours.

### Limitations

The main limitation of our analysis is the low quality of evidence, which can be explained, at least partially, by the non-randomized nature of most studies. In fact, a selection bias within most studies cannot be ruled out and it might be possible that healthier patients undergoing uncomplicated procedures were selected for SDD as compared with ONS.

Due to limited data, we could not evaluate the impact of population baseline differences and other potential residual confounders on pooled estimates.

Although we performed several sensitivity and subgroup analyses, we observed a significant degree of heterogeneity in outcomes’ estimates. However, this finding can be related to different study design, populations, and assessment of outcomes. Additionally, variations in SDD protocols or different pre-specified study- or centre-specific criteria regarding patient selection constitute another limitation as to the generalizability of the results. Due to different patient selection criteria across studies and exclusion of patients at higher risk of complications due to age or co-morbidities in many studies, subgroup analyses regarding patients in which SDD is not yet common (e.g. elderly or frail patients) or direct comparison of ablation techniques (e.g. RF, cryoablation, and PFA^57^) were not feasible. Another potential limitation of the current meta-analysis is that short-term complications after discharge may have been underestimated in the ONS cohort as some of them would have occurred during extended hospitalization compared with SDD. However, as other studies have shown that most complications after AF ablation occur during the first 6 h after the procedure, this potential limitation seems unlikely.

Finally, despite our rigorous search strategy and study evaluation process, it might be possible that some studies were not included.

Therefore, our findings should be interpreted with caution and as hypotheses generating.

## Conclusion

In this large meta-analysis of the current and most recent evidence on SDD after AF ablation, short-term and 30-day complications, re-hospitalization/ER visits, and 30-day mortality were rare and not elevated after SDD in comparison with ONS. Further large-scale prospective, randomized trials are needed in order to confirm safety of SDD after AF ablation and define patient-related eligibility criteria and optimal peri-SDD protocols.

## Supplementary Material

euae200_Supplementary_Data

## Data Availability

The data underlying this article are available in the article as well as in the online supplement material.
