# Supplementary material for: Same-day discharge vs. overnight stay following catheter ablation for atrial fibrillation: a comprehensive review and meta-analysis by the European Heart Rhythm Association Health Economics Committee
Source: Europace. 2024 Jul 30;26(8):euae200. doi: 10.1093/europace/euae200 (PMC11321359; doi:10.1093/europace/euae200)
Supplement: euae200_Supplementary_Data [file euae200_supplementary_data.docx]

**Supplementary Materials to:**

**Same Day Discharge vs. Overnight Stay Following Catheter Ablation for Atrial Fibrillation - A Comprehensive Review and Meta-Analysis by the EHRA Health Economics Committee**

Short Title: Same Day Discharge after Catheter Ablation of Atrial Fibrillation

**Maura M. Zylla,**^1,2,^ **Jacopo F. Imberti,**^3,4*^ **Francisco Leyva^2,5^, Ruben Casado-Arroyo^2,6^, Frieder Braunschweig ^2,7^, Helmut Pürerfellner ^8^, José L Merino ^9^, Giuseppe Boriani^2,3^**

^1^Department of Cardiology, Heidelberg Center of Heart Rhythm Disorders, Medical University Hospital, Im Neuenheimer Feld 410, Heidelberg, Germany;

^2^Health Economics Committee of EHRA (European Heart Rhythm Association);

^3^Cardiology Division, Department of Biomedical, Metabolic and Neural Sciences, University of Modena and Reggio Emilia, Policlinico di Modena, Modena, Italy;

^4^Clinical and Experimental Medicine PhD Program, University of Modena and Reggio Emilia, Modena, Italy;

^5^Aston Medical Research Institute, Aston Medical School, Aston University, Aston Triangle, Birmingham B4 7ET, United Kingdom;

^6^Department of Cardiology, H.U.B.-Hôpital Erasme, Université Libre de Bruxelles, 1070 Brussels, Belgium;

^7^Department of Medicine; Solna, Karolinska Institutet and ME Cardiology, Karolinska University Hospital, Norrbacka S1:02, Eugeniavagen 27, 171 77 Stockholm, Sweden

^8^Ordensklinikum Linz Elisabethinen, Linz, Austria;

^9^Arrhythmia-Robotic Electrophysiology Unit, La Paz University Hospital, IdiPAZ, Universidad Autonoma, Madrid, Spain

*=Corresponding author:

Jacopo F Imberti,

Cardiology Division, Department of Biomedical, Metabolic and Neural Sciences

University of Modena and Reggio Emilia, Policlinico di Modena

Campus Scientifico

Edificio Scienze Biomediche

Via Giuseppe Campi, 287

41125 Modena, Italy

Tel.: +39 059 4225836.

E-Mail : [jacopo.imberti@hotmail.it](mailto:jacopo.imberti@hotmail.it)

**Supplementary Figure 1.** Pooled prevalence of short-term complications after same day discharge.

**Supplementary Figure 2.** Leave-one-out analysis for pooled prevalence of short-term complications after same day discharge.

**Supplementary Figure 3.** Subgroup analysis for pooled prevalence of short-term complications after same day discharge.

**Supplementary Figure 4.** Pooled prevalence of 30-days complications after same day discharge.

**Supplementary Figure 5.** Leave-one-out analysis for pooled prevalence of 30-days complications after same day discharge.

**Supplementary Figure 6.** Subgroup analysis for pooled prevalence of 30-days complications after same day discharge.

**Supplementary Figure 7.** Leave-one-out analysis for risk ratio of 30-days complications of same day discharge strategy versus overnight stay.

**Supplementary Figure 8.** Subgroup analysis for risk ratio of 30-days complications of same day discharge strategy versus overnight stay. **Panel A** shows prospective vs retrospective studies. **Panel B** shows administrative vs non administrative studies.

**Supplementary Figure 9.** Pooled prevalence of 30-days mortality after same day discharge.

**Supplementary Figure 10.** Leave-one-out analysis for pooled prevalence of 30-days mortality after same day discharge.

**Supplementary Figures 11.** Subgroup analysis for pooled prevalence of 30-days mortality after same day discharge.

**Supplementary Figures 12.** Leave-one-out analysis for risk ratio of 30-days mortality of same day discharge strategy versus overnight stay.

**Supplementary Figures 13.** Subgroup analysis for risk ratio of 30-days mortality of same day discharge strategy versus overnight stay.

**Supplemental Figure 14.** Pooled prevalence of unplanned medical contact at 30 days post-discharge

after same day discharge.

**Supplementary Figure 15.** Leave-one-out analysis for pooled prevalence of unplanned medical contact at 30 days post-discharge after same day discharge.

**Supplementary Figure 16.** Subgroup analysis for pooled prevalence of unplanned medical contact at 30 days post-discharge after same day discharge.

**Supplementary Figure 17.** Leave-one-out analysis for risk ratio of unplanned medical contact at 30 days post-discharge after same day discharge.

**Supplementary Figure 18.** Subgroup analysis for risk ratio of unplanned medical contact at 30 days post-discharge after same day discharge. Prospective vs retrospective data.

**Supplementary Figure 19.** Subgroup analysis for risk ratio of unplanned medical contact at 30 days post-discharge after same day discharge. SDD as default strategy vs non-default strategy.

**Supplementary Figure 20.** Funnel plots for publication bias: pooled prevalence of 30 days complications.

**Supplementary Figure 21.** Funnel plots for publication bias: risk ratio of 30 days complications.

**Supplementary Figure 22.** Funnel plots for publication bias: pooled prevalence of 30 days mortality.

**Supplementary Figure 23.** Funnel plots for publication bias: pooled prevalence of unplanned medical contact at 30 days.

**Supplementary Table 1.** Newcastle-Ottawa quality assessment for non-randomized studies.

**Supplementary Figure 24.** Risk of bias assessment using the algorithm in RoB2 for randomized controlled trials.

**Supplementary Table 2.** PRISMA checklist.

**Supplementary Figure 1.** Pooled prevalence of short-term complications after same day discharge.

**
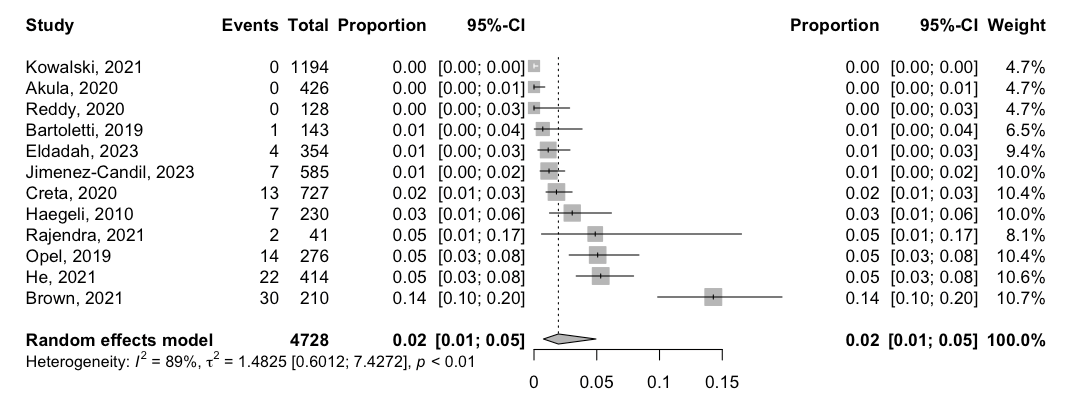
**

**Supplementary Figure 2.** Leave-one-out analysis for pooled prevalence of short-term complications after same day discharge.

**
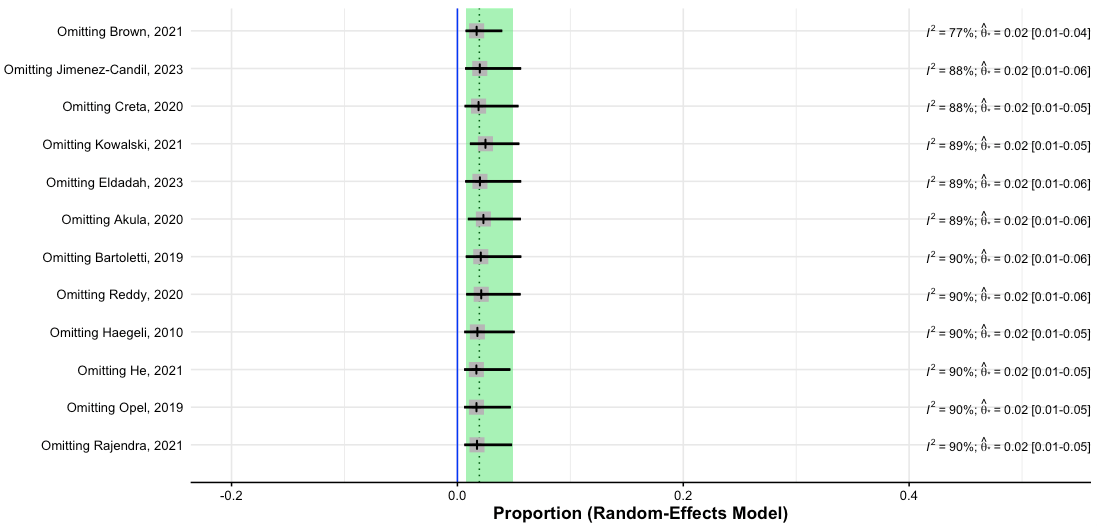
**

**Supplementary Figure 3.** Subgroup analysis for pooled prevalence of short-term complications after same day discharge.

**
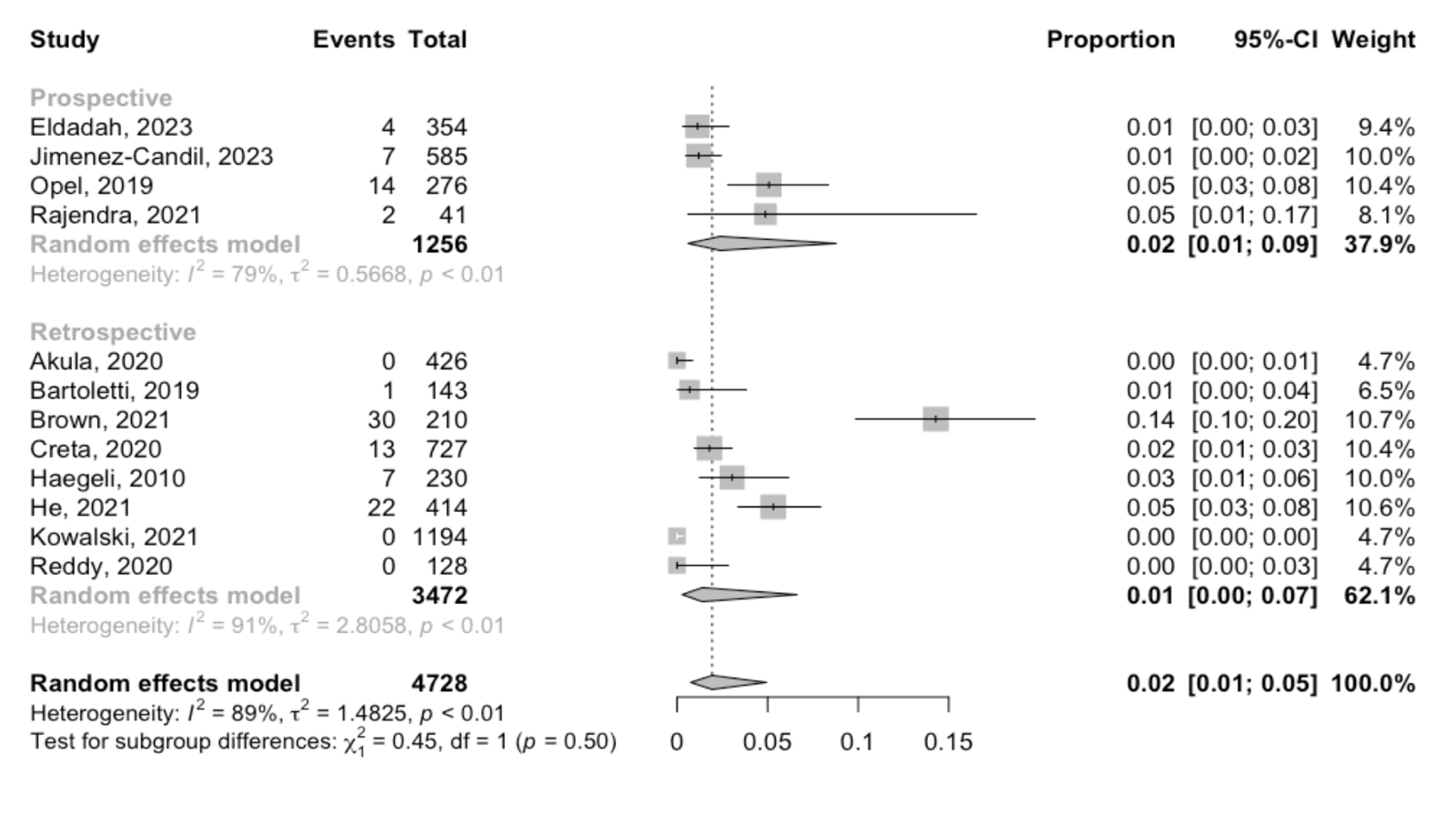
**

**Supplementary Figure 4.** Pooled prevalence of 30-days complications after same day discharge.

**
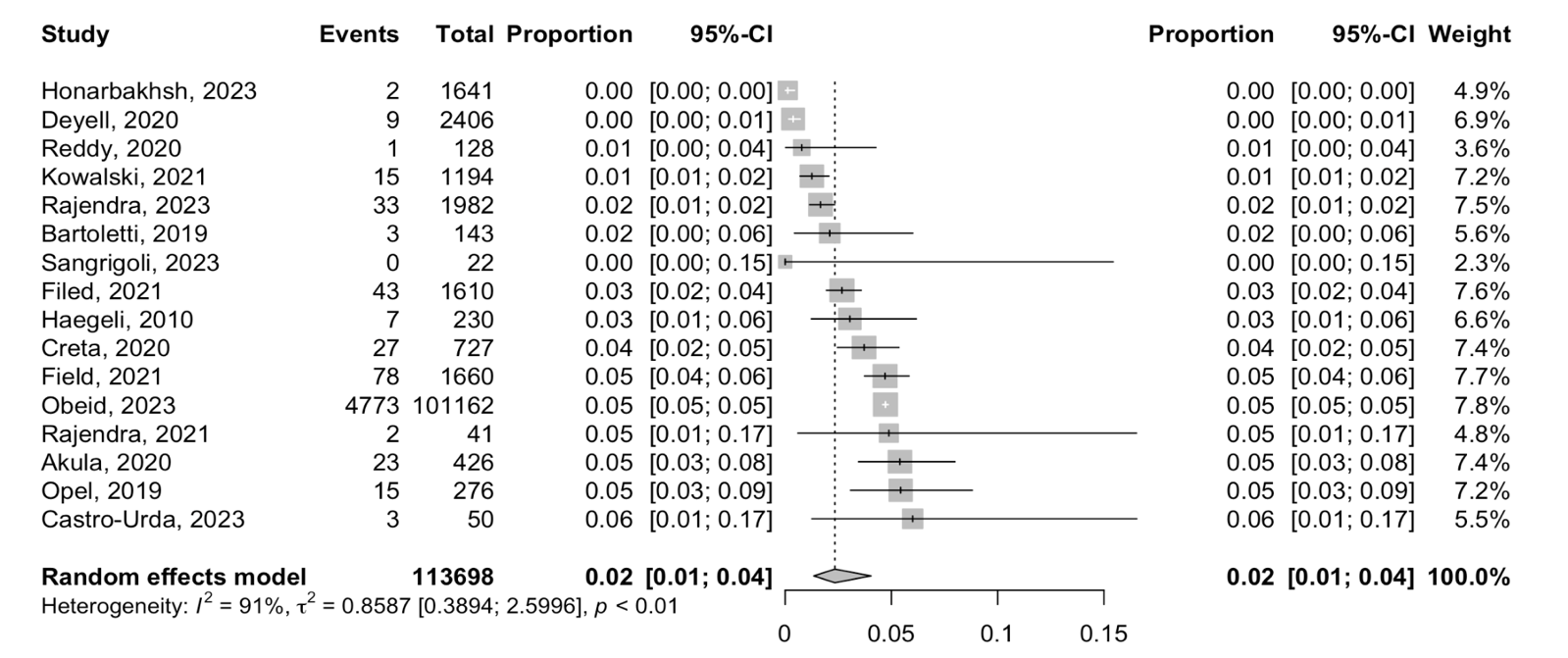
**

**Supplementary Figure 5.** Leave-one-out analysis for pooled prevalence of 30-days complications after same day discharge.

**
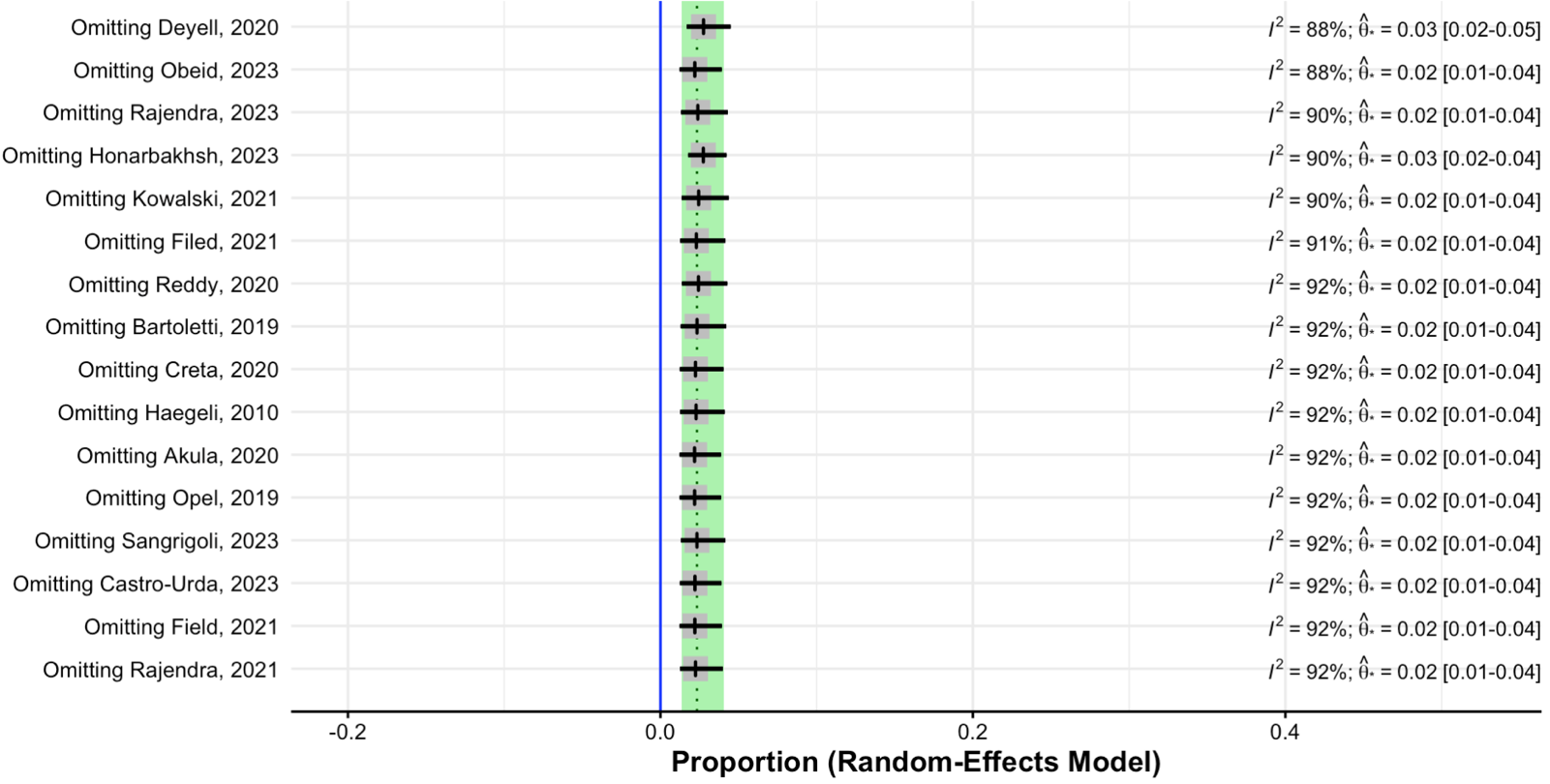
**

**Supplementary Figure 6.** Subgroup analysis for pooled prevalence of 30-days complications after same day discharge.

**
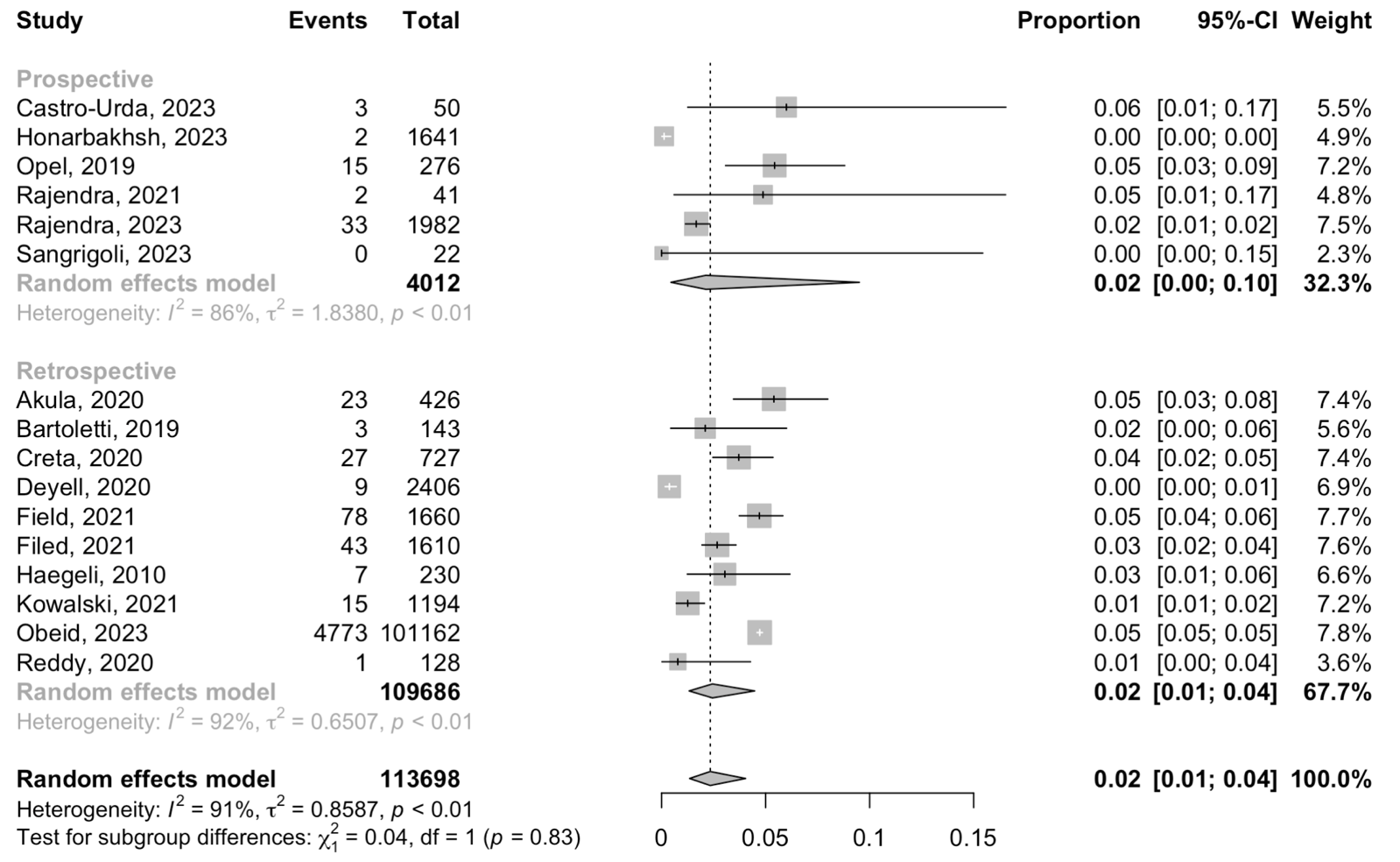
**

**Supplementary Figure 7.** Leave-one-out analysis for risk ratio of 30-days complications of same day discharge strategy versus overnight stay.

**
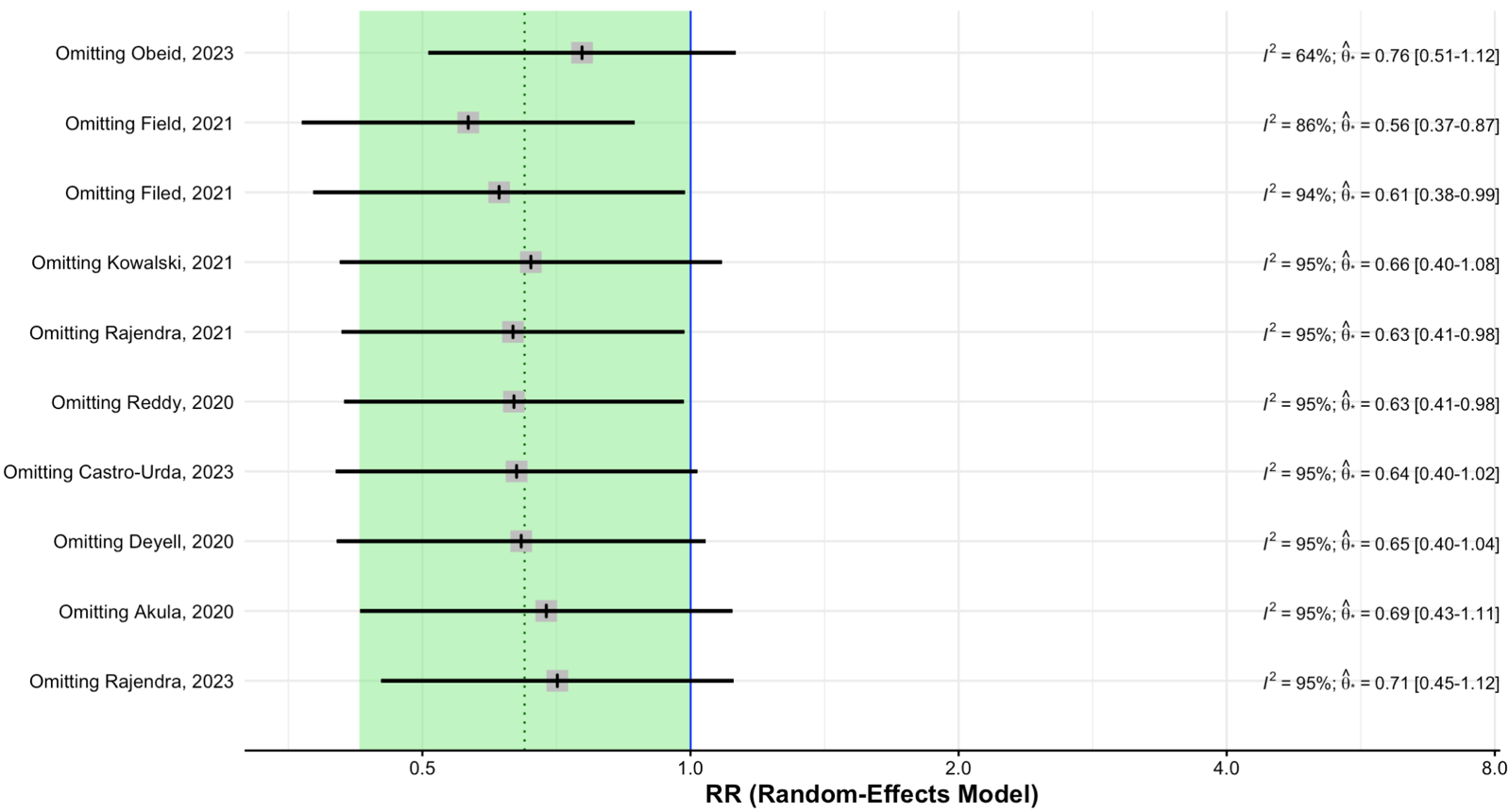
**

**Supplementary Figure 8.** Subgroup analysis for risk ratio of 30-days complications of same day discharge strategy versus overnight stay. **Panel A** shows prospective vs retrospective studies. **Panel B** shows administrative vs non administrative studies.

**
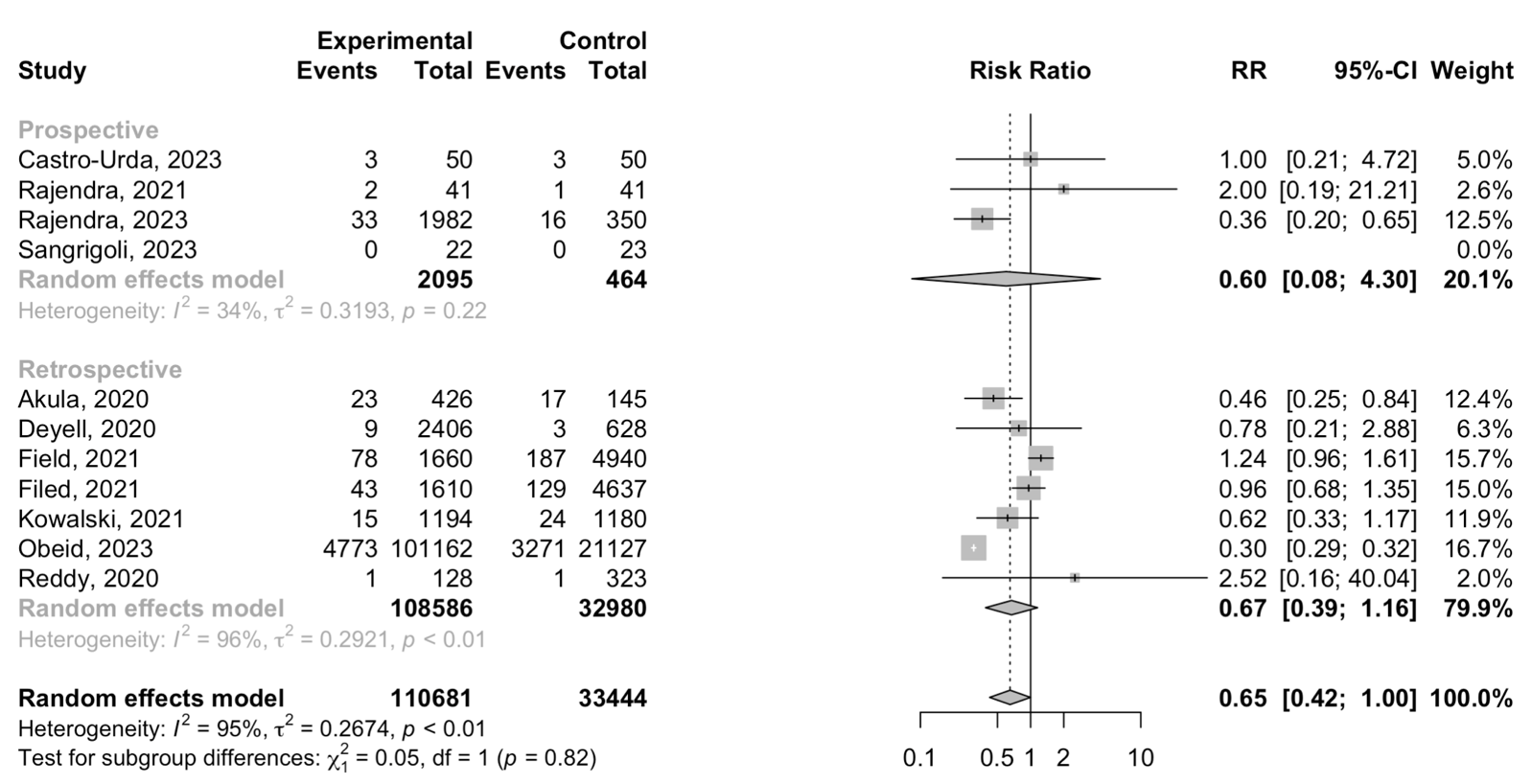
**

**A**

**
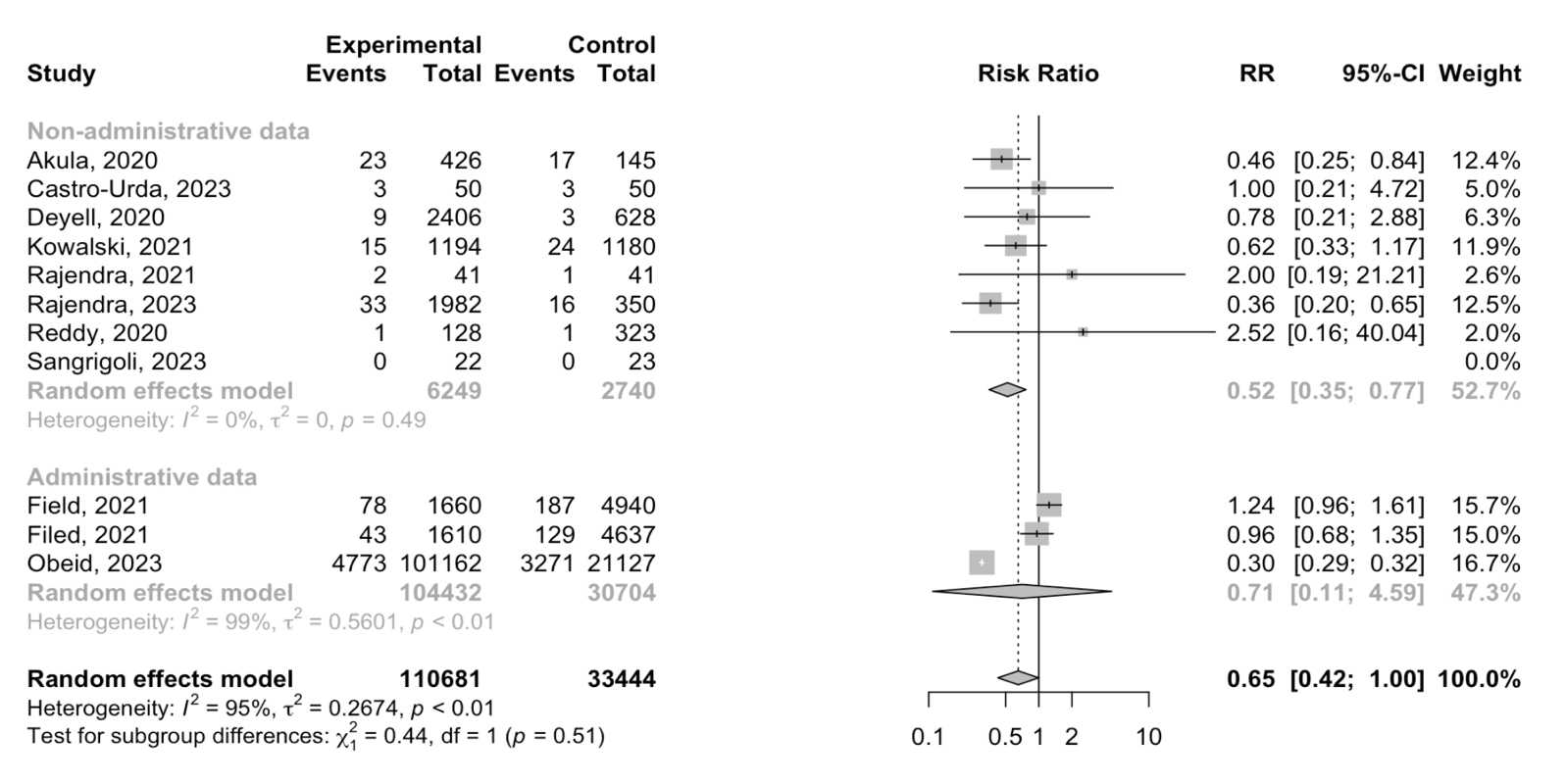
**

**B**

**Supplementary Figure 9.** Pooled prevalence of 30-days mortality after same day discharge.

**
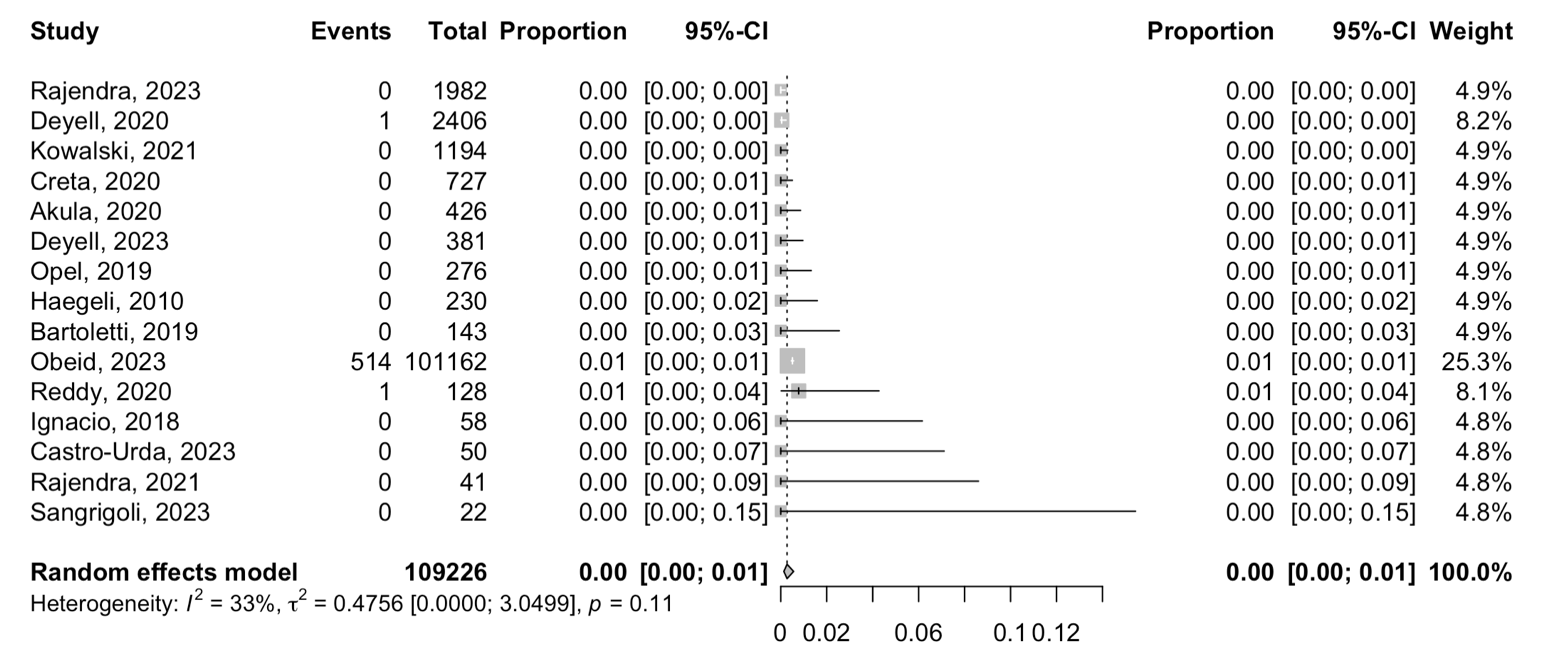
**

**Supplementary Figure 10.** Leave-one-out analysis for pooled prevalence of 30-days mortality after same day discharge.

**
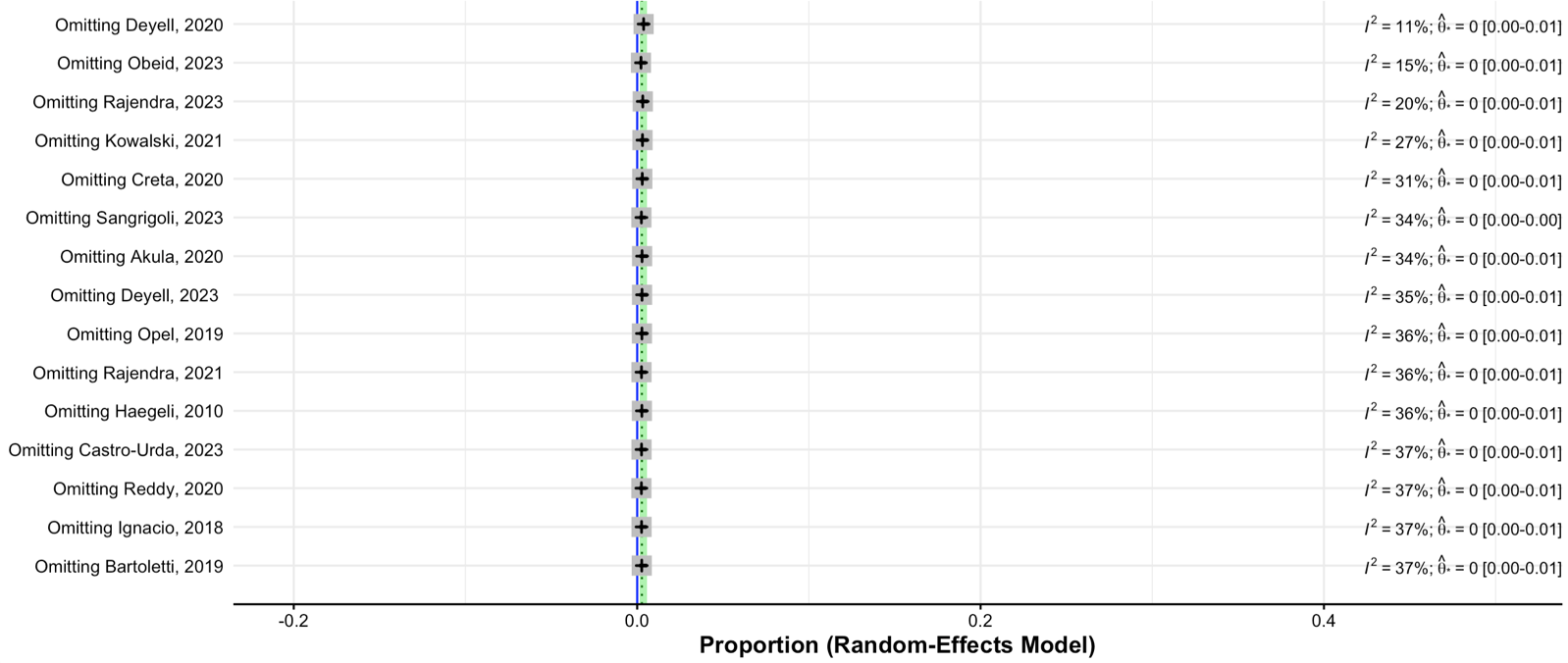
**

**Supplementary Figure 11.** Subgroup analysis for pooled prevalence of 30-days mortality after same day discharge.

**
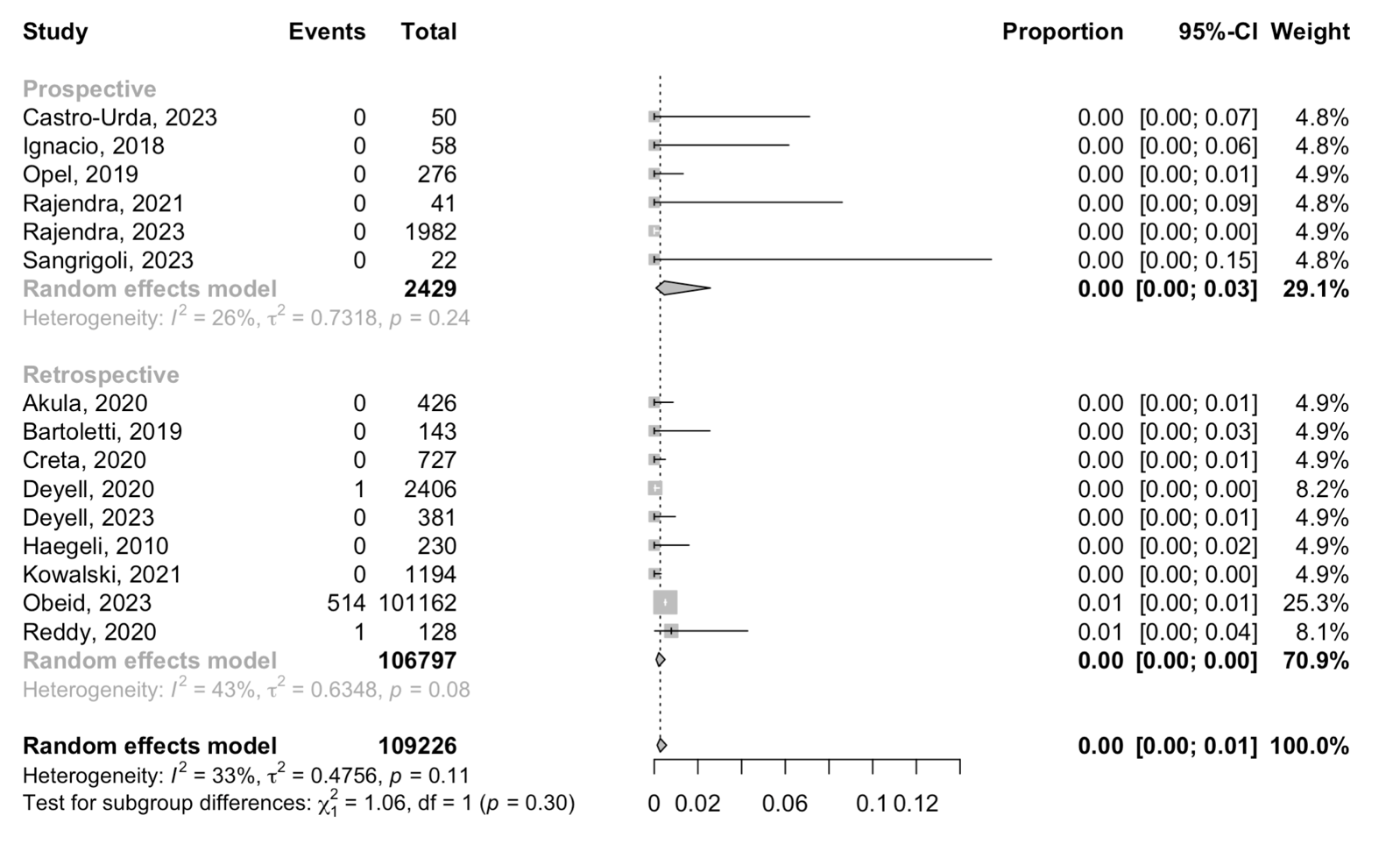
**

**Supplementary Figure 12.** Leave-one-out analysis for risk ratio of 30-days mortality of same day discharge strategy versus overnight stay.

**
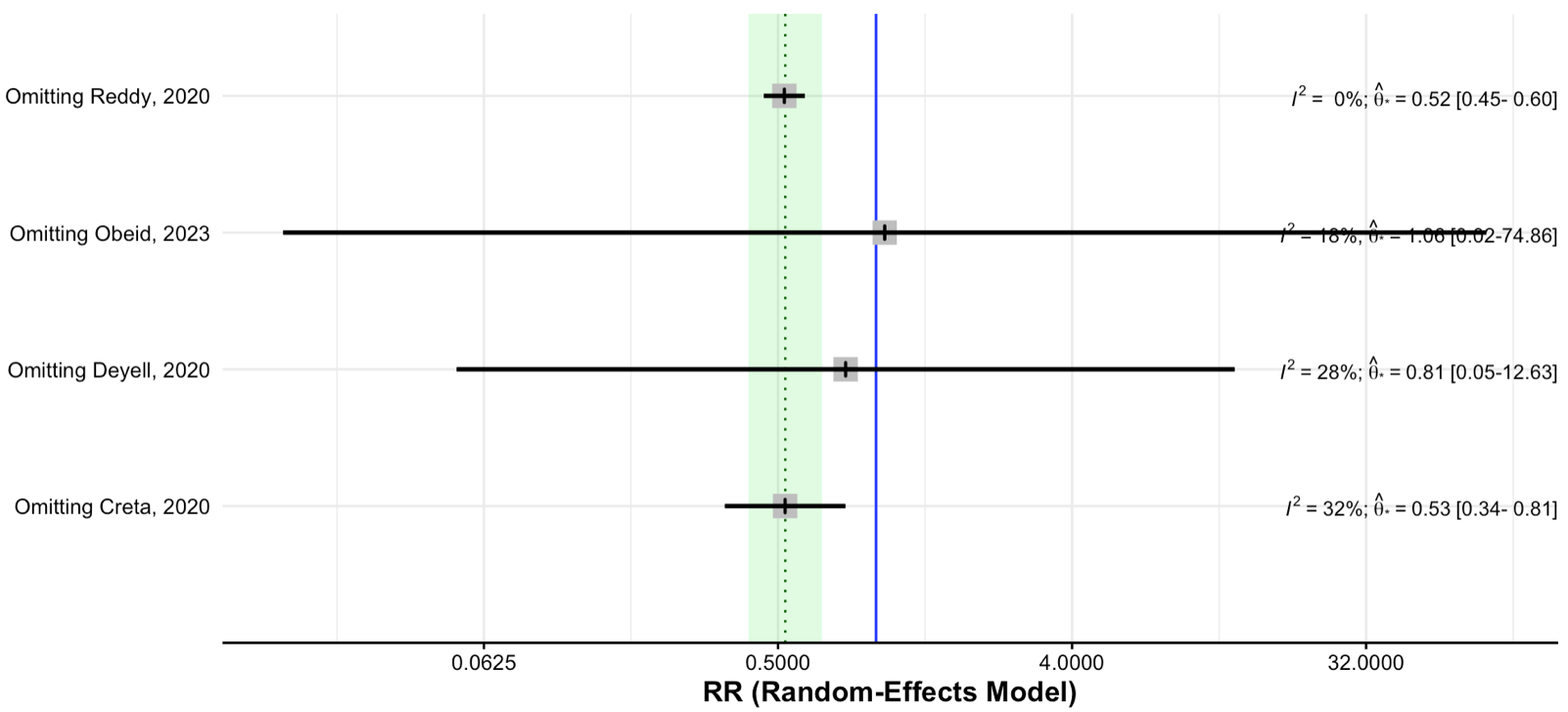
**

**Supplementary Figure 13.** Subgroup analysis for risk ratio of 30-days mortality of same day discharge strategy versus overnight stay.

**
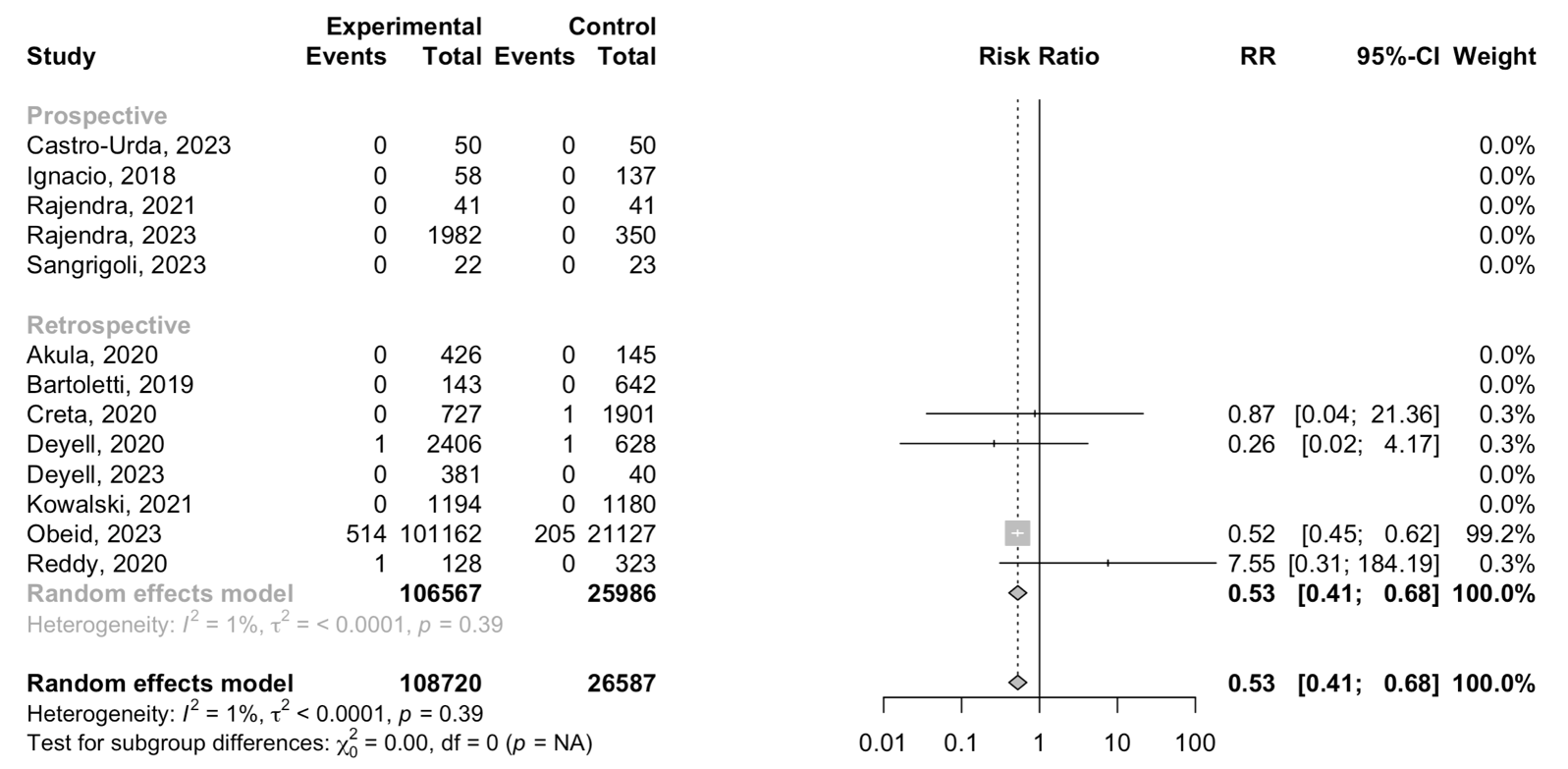
**

**Supplementary Figure 14.** Pooled prevalence of unplanned medical contact at 30 days post-discharge after same day discharge.

**
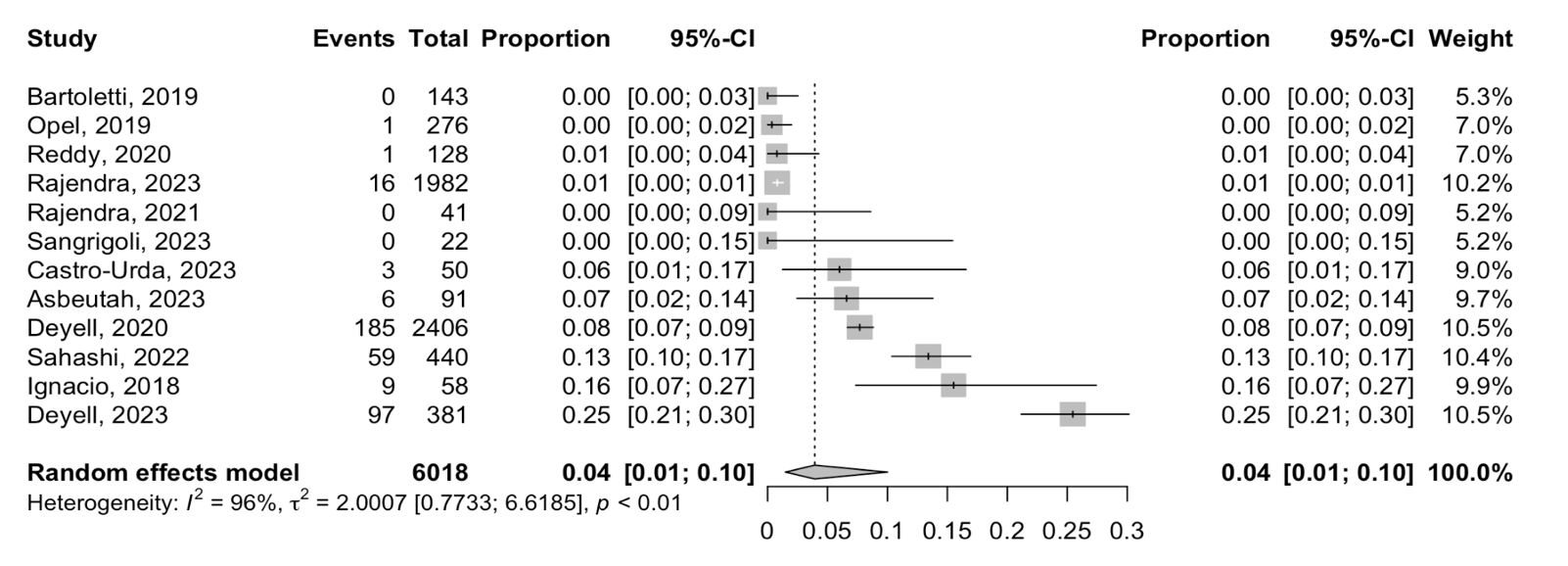
**

**Supplementary Figure 15.** Leave-one-out analysis for pooled prevalence of unplanned medical contact at 30 days post-discharge after same day discharge.

**
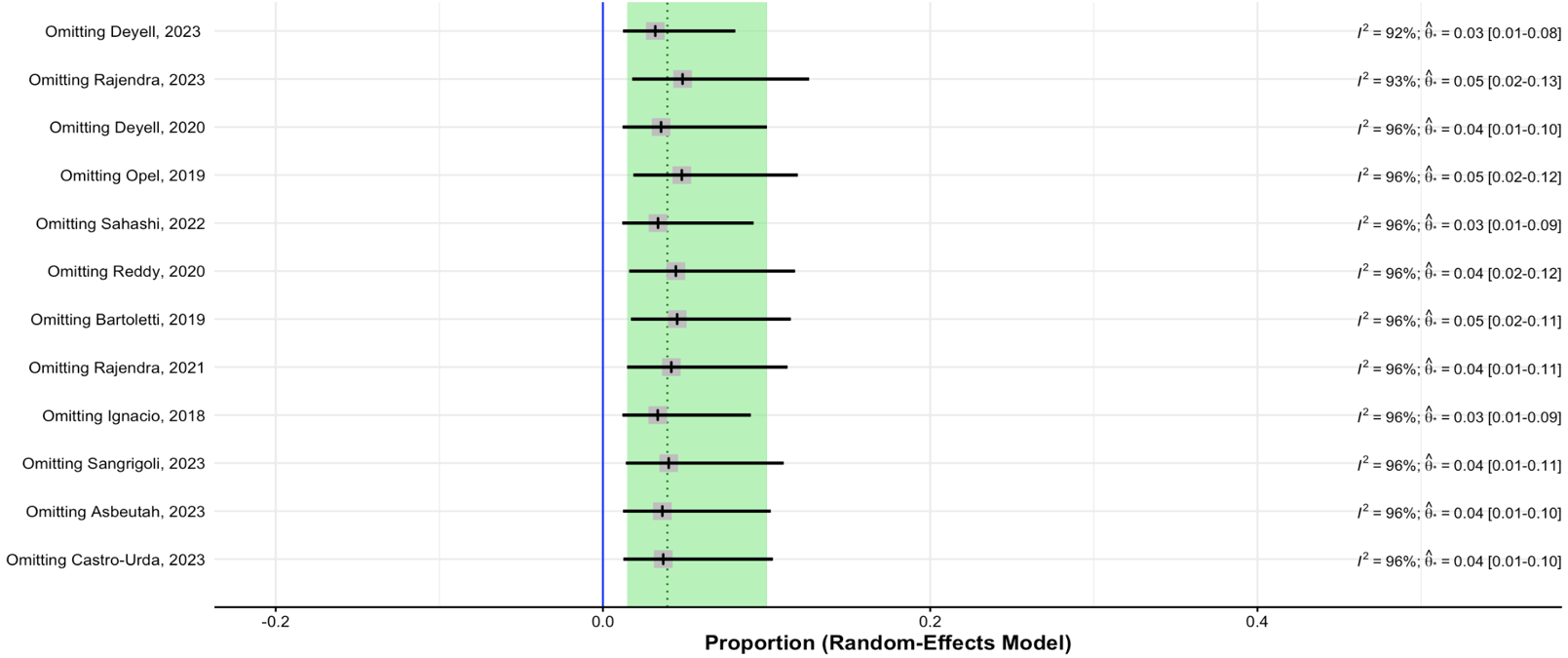
**

**Supplementary Figure 16.** Subgroup analysis for pooled prevalence of unplanned medical contact at 30 days post-discharge after same day discharge.

**
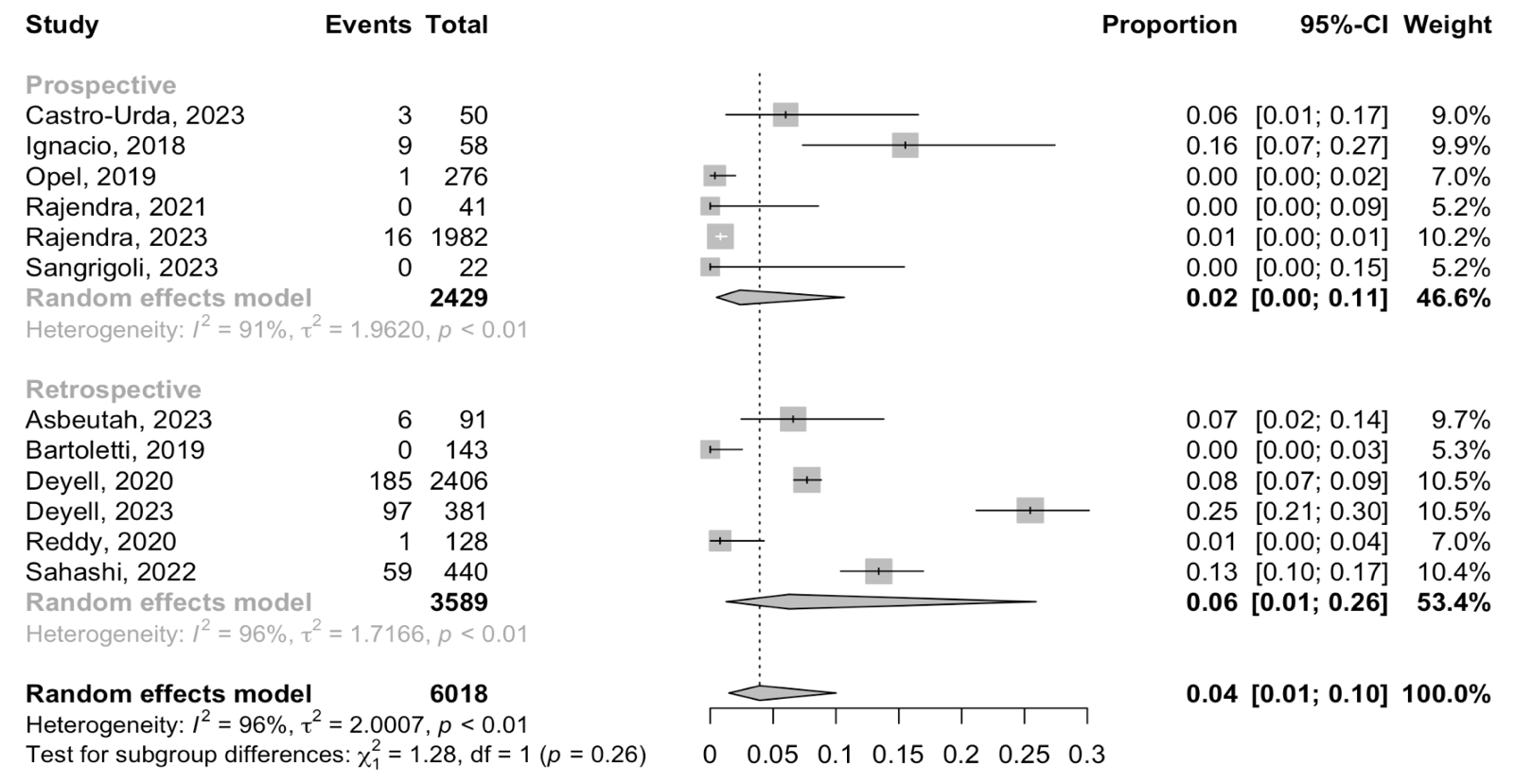
**

**Supplementary Figure 17.** Leave-one-out analysis for risk ratio of unplanned medical contact at 30 days post-discharge after same day discharge.

**
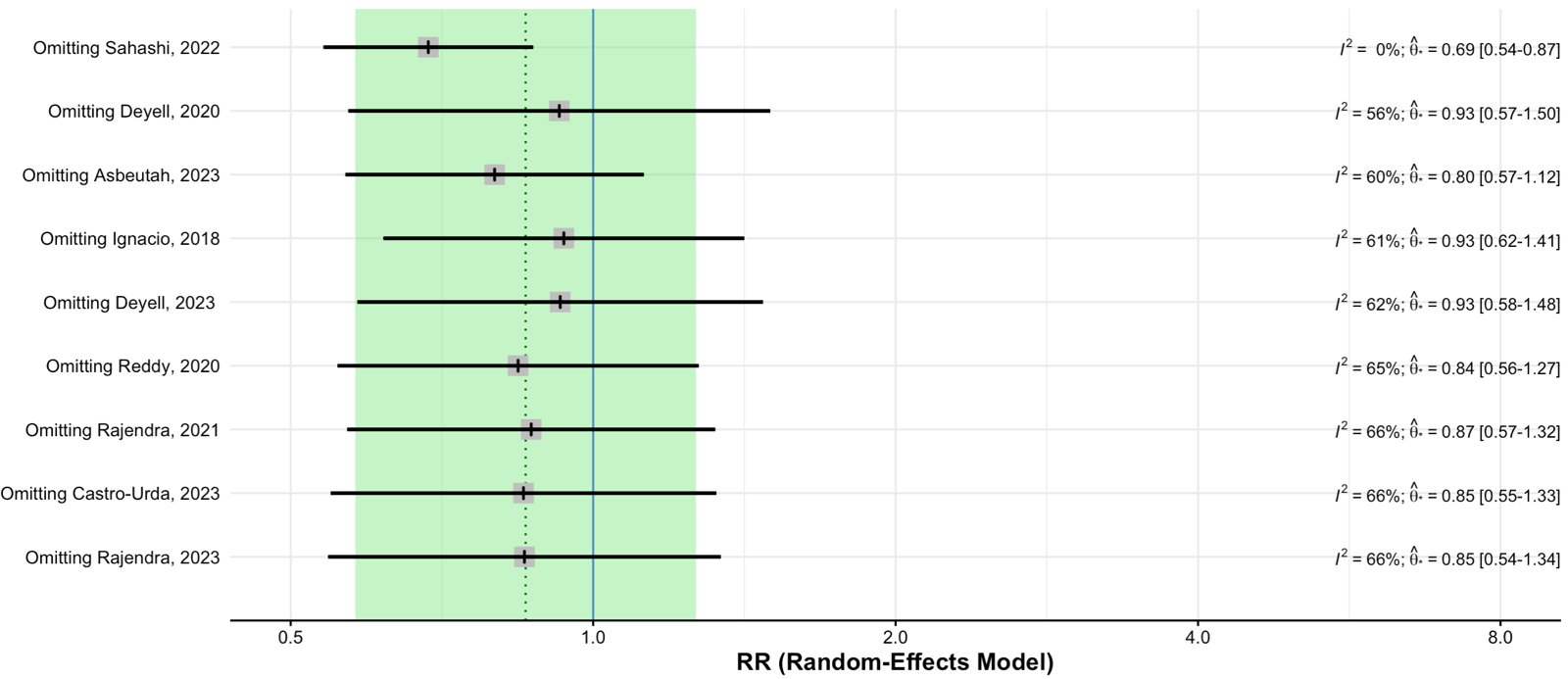
**

**Supplementary Figure 18.** Subgroup analysis for risk ratio of unplanned medical contact at 30 days post-discharge after same day discharge. Prospective vs retrospective data.

**
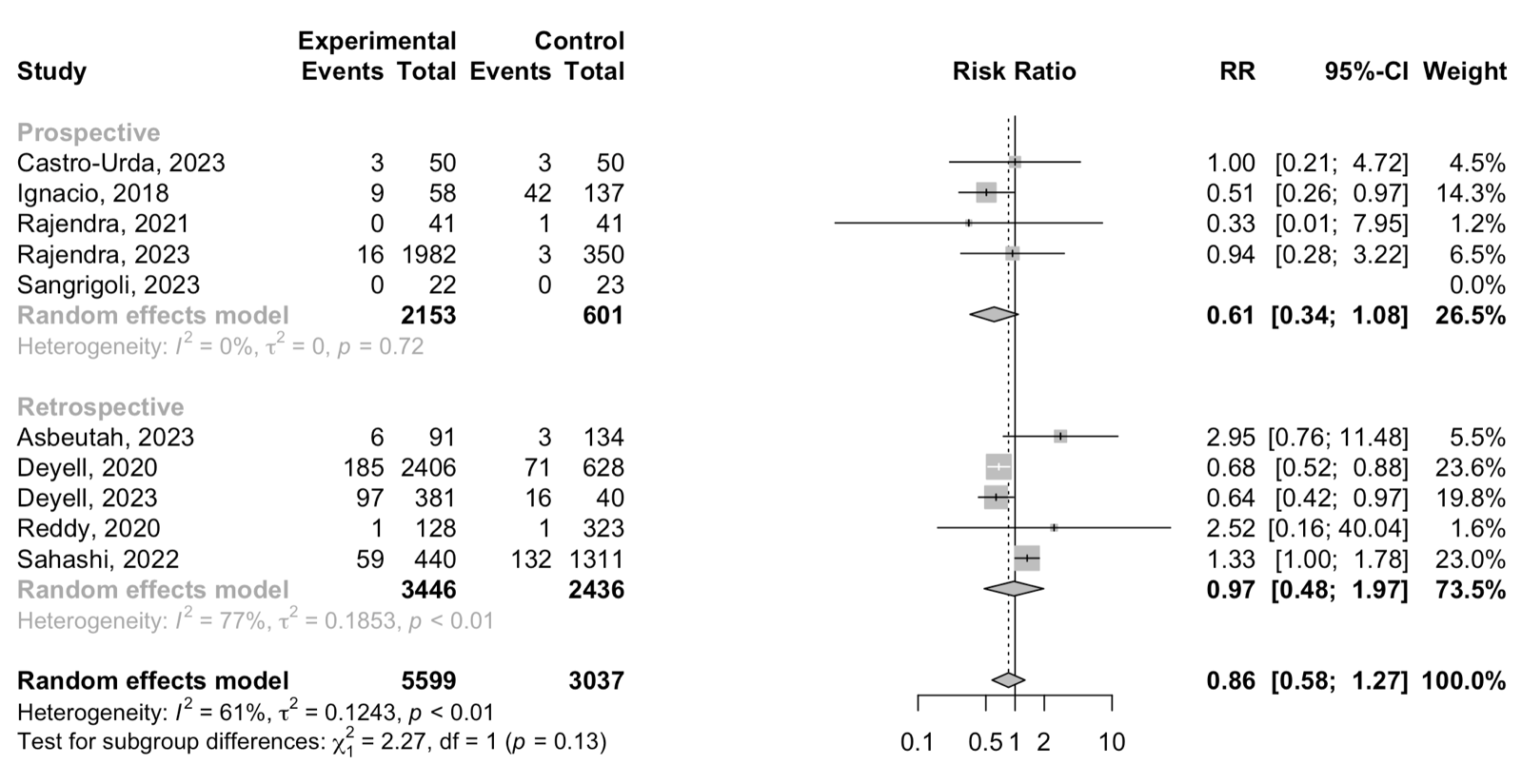
**

**Supplementary Figure 19.** Subgroup analysis for risk ratio of unplanned medical contact at 30 days post-discharge after same day discharge. SDD as default strategy vs non-default strategy.


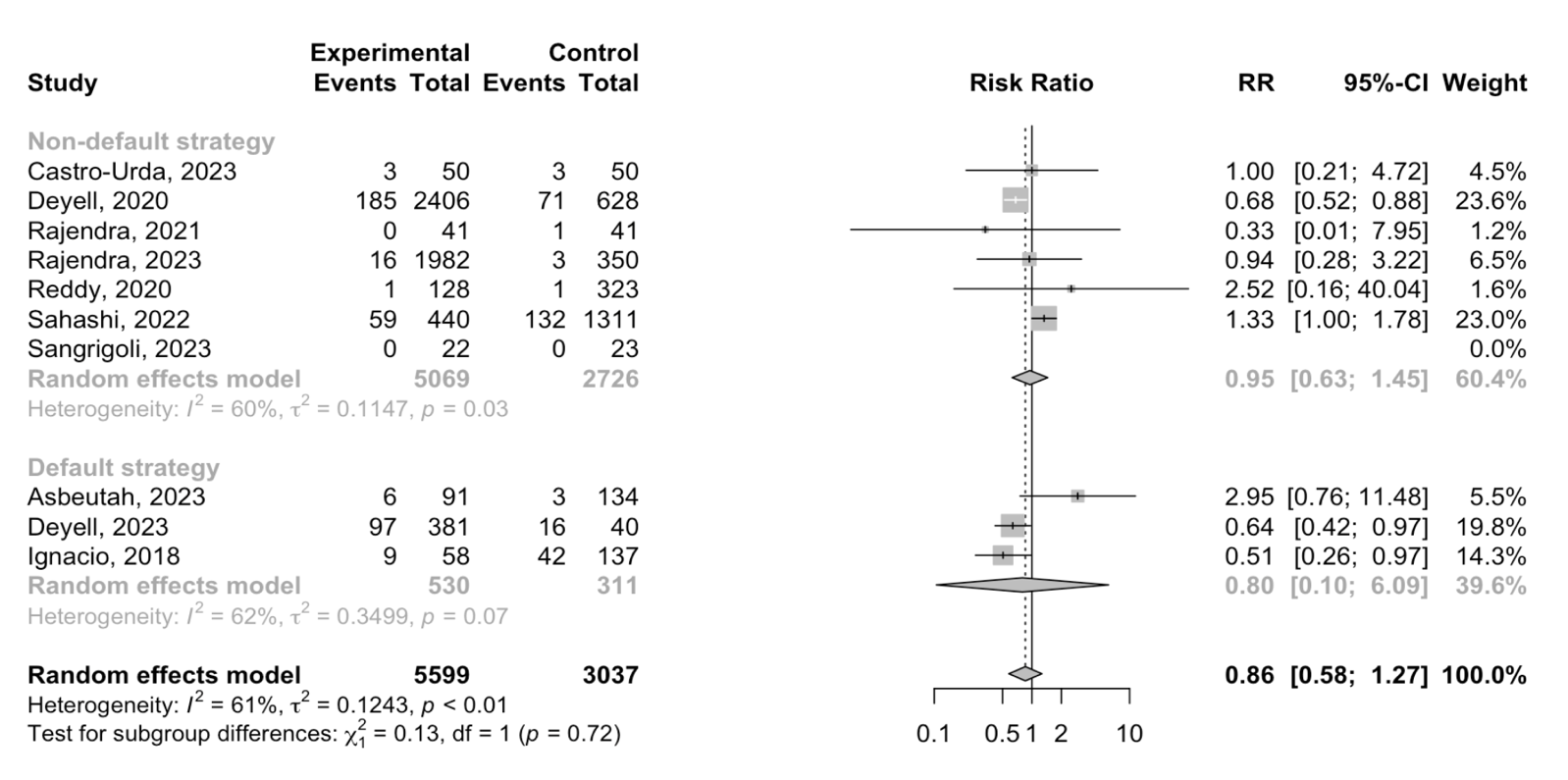


**Supplementary Figure 20.** Funnel plots for publication bias: pooled prevalence of 30 days complications.

Eggers’ test p-value= 0.009

**
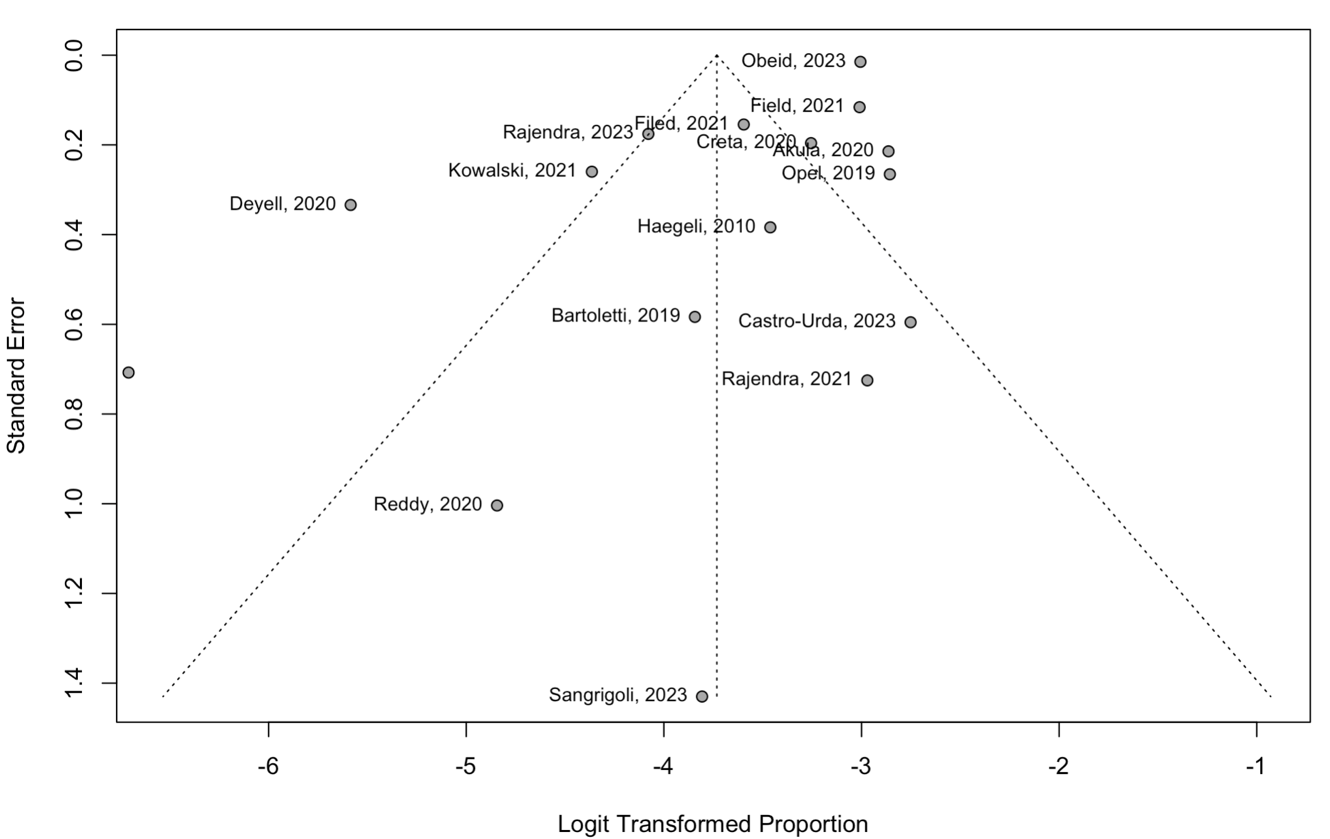
**

**Supplementary Figure 21.** Funnel plots for publication bias: risk ratio of 30 days complications.

Eggers’ test p-value=0.043

**
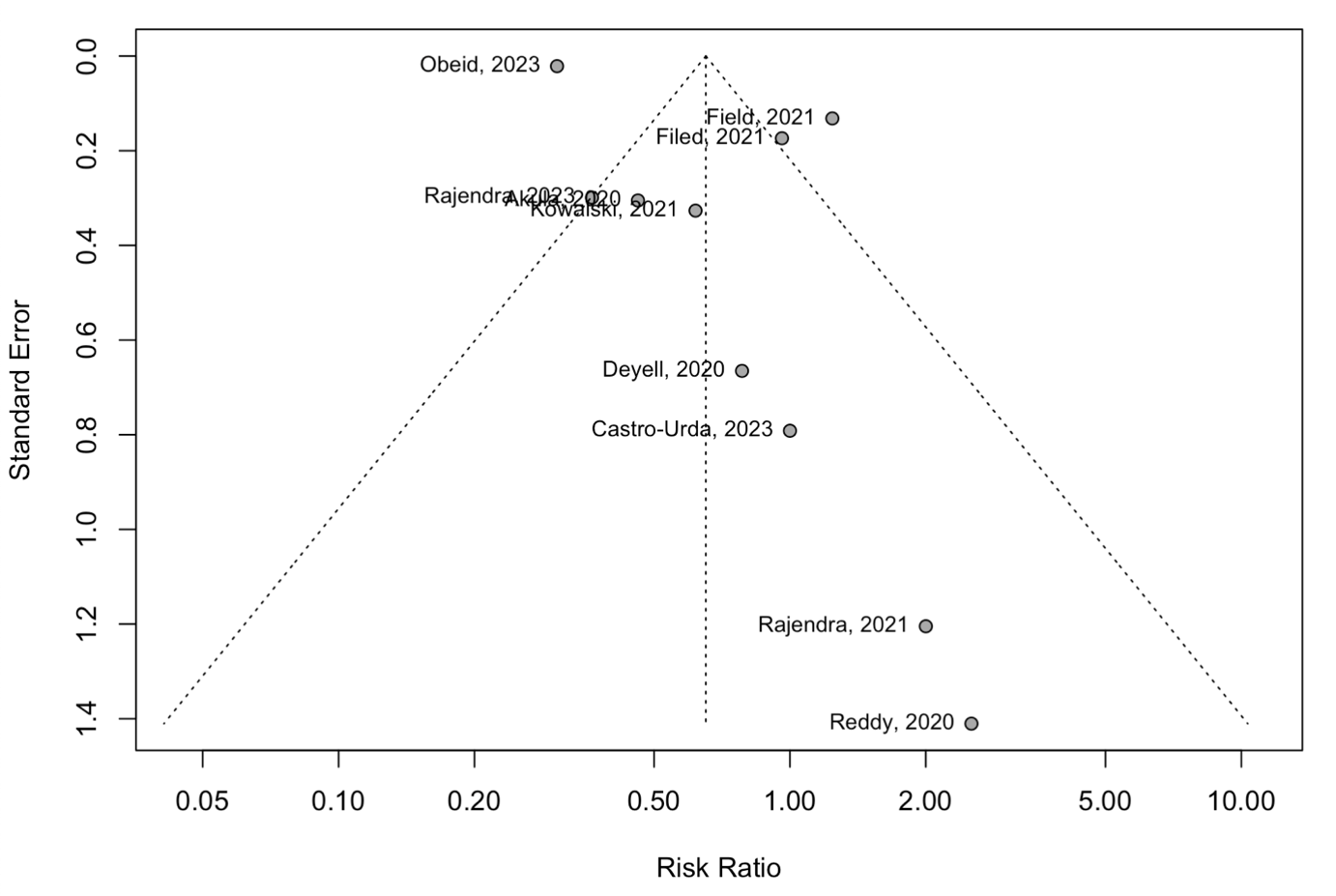
**

**Supplementary Figure 22.** Funnel plots for publication bias: pooled prevalence of 30 days mortality.

Eggers’ test p-value=0.060

**
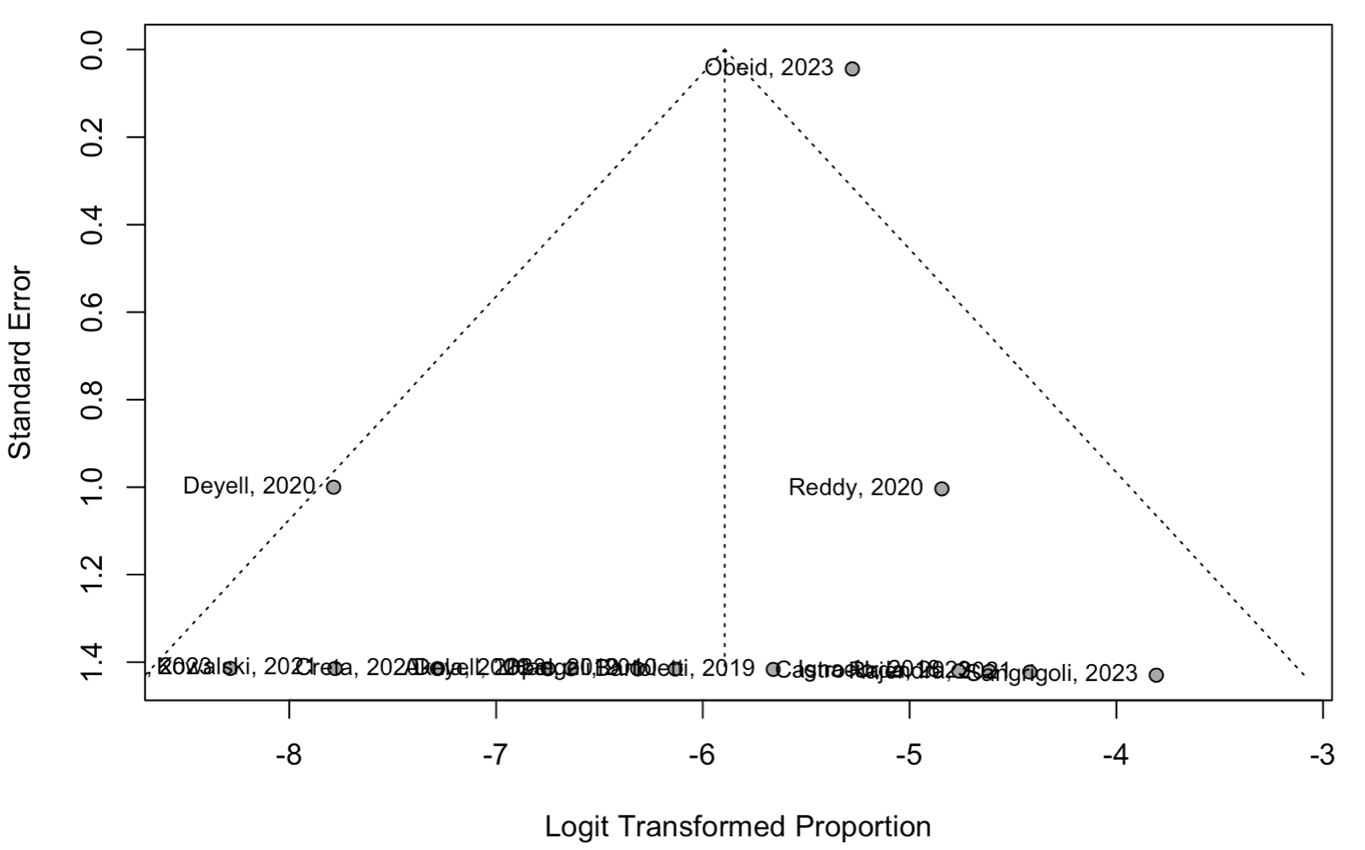
**

**Supplementary Figure 23.** Funnel plots for publication bias: pooled prevalence of unplanned medical contact at 30 days.

Eggers’ test p-value=0.269

**
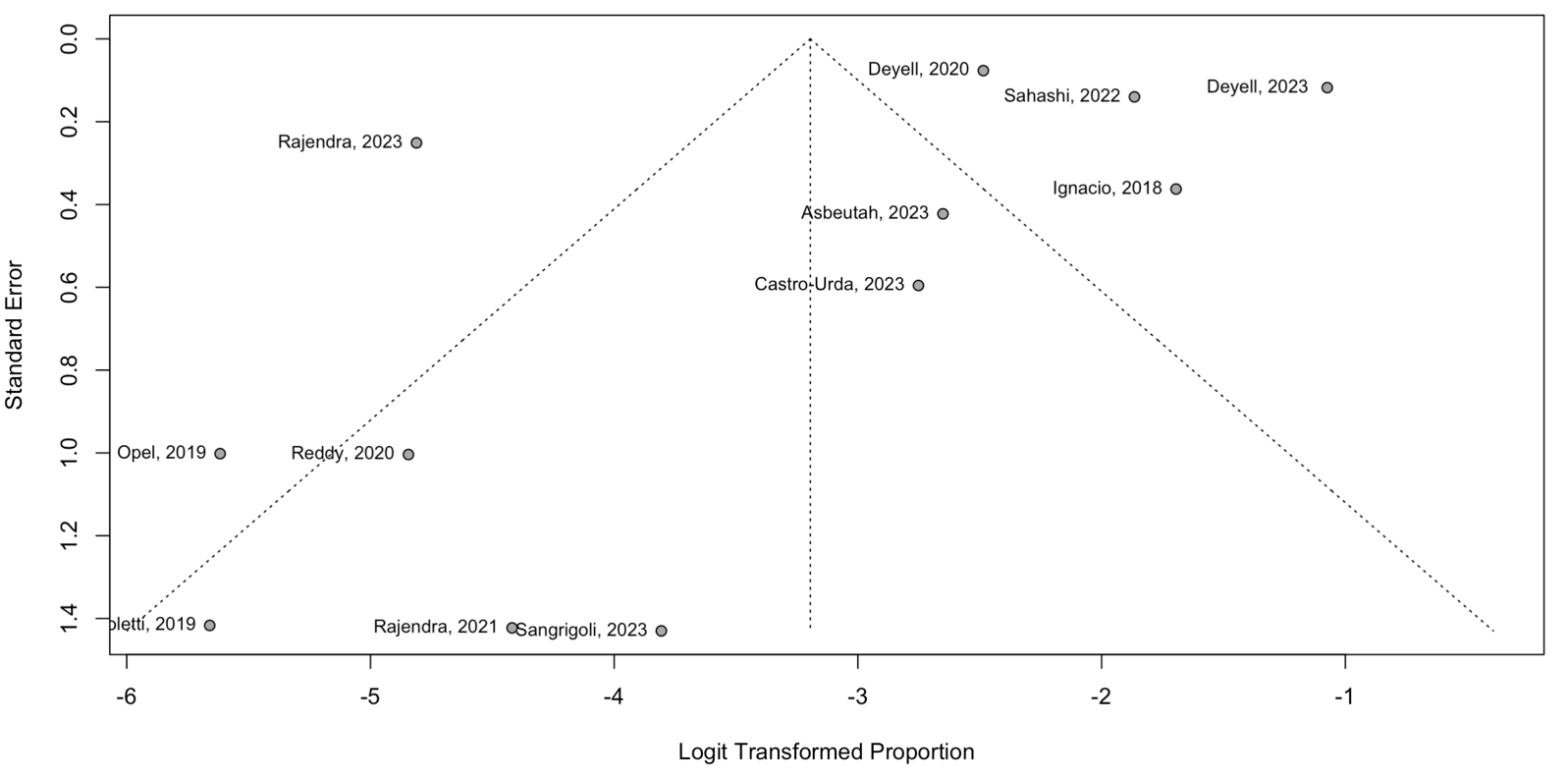
**

**Supplementary Table 1.** Newcastle-Ottawa quality assessment for non-randomized studies.

|  | **Representativeness of exposed cohort** | **Selection of the non-exposed cohort** | **Ascertain-ment of exposure** | **Outcome of interest not present at start** | **Comparability** | **Assessment of outcome** | **Follow-up long enough** | **Adequacy of follow up** | **Total** |
| --- | --- | --- | --- | --- | --- | --- | --- | --- | --- |
| Haegeli et al., 2010 | 1 | 0 | 0 | 1 | 0 | 0 | 1 | 1 | 4 |
| Ignacio et al., 2018 | 1 | 1 | 1 | 1 | 0 | 0 | 1 | 1 | 6 |
| Bartoletti et al., 2019 | 1 | 1 | 1 | 1 | 0 | 1 | 1 | 1 | 7 |
| Opel et al., 2019 | 1 | 1 | 1 | 1 | 0 | 1 | 1 | 1 | 7 |
| Creta et al., 2020 | 1 | 0 | 1 | 1 | 0 | 1 | 1 | 1 | 6 |
| Akula et al., 2020 | 1 | 1 | 1 | 1 | 0 | 1 | 1 | 1 | 7 |
| Reddy et al., 2020 | 1 | 1 | 1 | 1 | 0 | 1 | 1 | 1 | 7 |
| Deyell et al., 2020 | 1 | 1 | 1 | 1 | 0 | 1 | 1 | 1 | 7 |
| Brown et al., 2021 | 1 | 0 | 0 | 1 | 0 | 0 | 1 | 1 | 4 |
| Field et al., 2021 | 1 | 0 | 1 | 1 | 2 | 1 | 1 | 0 | 7 |
| Field et al., 2021 | 1 | 0 | 1 | 1 | 2 | 1 | 1 | 0 | 7 |
| Kowalski et al., 2021 | 1 | 0 | 1 | 1 | 1 | 1 | 1 | 1 | 7 |
| Rajendra et al., 2021 | 1 | 0 | 1 | 1 | 1 | 0 | 1 | 1 | 6 |
| He et al., 2021 | 1 | 1 | 1 | 1 | 0 | 1 | 1 | 1 | 7 |
| Sahashi et al., 2022 | 1 | 0 | 1 | 1 | 2 | 1 | 1 | 0 | 7 |
| Rajendra et al., 2023 | 1 | 0 | 1 | 1 | 0 | 1 | 1 | 1 | 6 |
| Asbeutah et al., 2023 | 1 | 1 | 1 | 1 | 0 | 1 | 1 | 1 | 7 |
| Obeid et al., 2023 | 1 | 0 | 1 | 1 | 1 | 1 | 1 | 1 | 7 |
| Eldadah et al., 2023 | 1 | 1 | 1 | 1 | 0 | 1 | 1 | 1 | 7 |
| Deyell et al., 2023 | 1 | 0 | 1 | 1 | 0 | 1 | 1 | 1 | 6 |
| Jimenez-Candil et al., 2023 | 1 | 0 | 1 | 1 | 0 | 1 | 1 | 1 | 6 |
| Honarbakhsh et al., 2023 | 1 | 0 | 0 | 1 | 1 | 0 | 1 | 1 | 5 |

**Supplementary Figure 24.** Risk of bias assessment using the algorithm in RoB2 for randomized controlled trials.

**
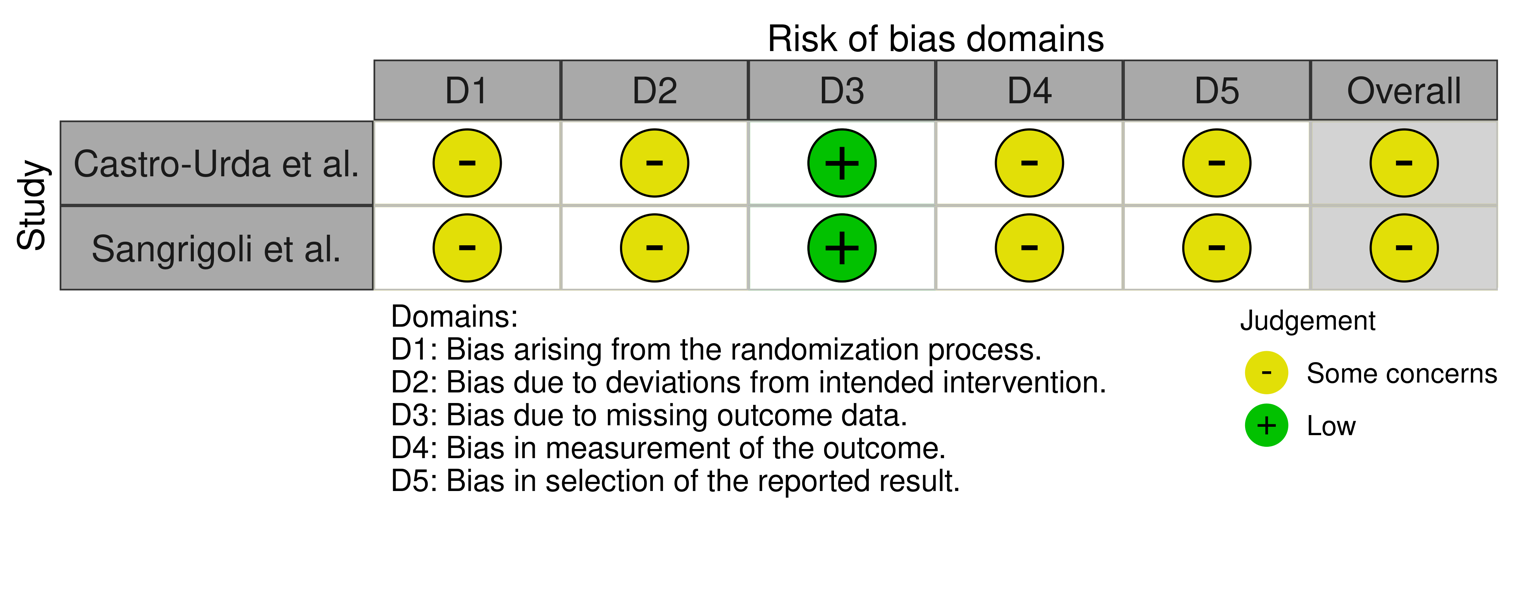
**

**Supplementary Table 2.** PRISMA checklist.

| **Section and Topic** | **Item #** | **Checklist item** | **Location where item is reported** |
| --- | --- | --- | --- |
| **TITLE** | | |  |
| Title | 1 | Identify the report as a systematic review. | Pages 1, 3, 5,6 |
| **ABSTRACT** | | |  |
| Abstract | 2 | See the PRISMA 2020 for Abstracts checklist. | Page 3 |
| **INTRODUCTION** | | |  |
| Rationale | 3 | Describe the rationale for the review in the context of existing knowledge. | Page 5 |
| Objectives | 4 | Provide an explicit statement of the objective(s) or question(s) the review addresses. | Page 5/6 |
| **METHODS** | | |  |
| Eligibility criteria | 5 | Specify the inclusion and exclusion criteria for the review and how studies were grouped for the syntheses. | Page 6-8 |
| Information sources | 6 | Specify all databases, registers, websites, organisations, reference lists and other sources searched or consulted to identify studies. Specify the date when each source was last searched or consulted. | Page 6 |
| Search strategy | 7 | Present the full search strategies for all databases, registers and websites, including any filters and limits used. | Page 6 |
| Selection process | 8 | Specify the methods used to decide whether a study met the inclusion criteria of the review, including how many reviewers screened each record and each report retrieved, whether they worked independently, and if applicable, details of automation tools used in the process. | Pages 6/7 |
| Data collection process | 9 | Specify the methods used to collect data from reports, including how many reviewers collected data from each report, whether they worked independently, any processes for obtaining or confirming data from study investigators, and if applicable, details of automation tools used in the process. | Pages 6/7 |
| Data items | 10a | List and define all outcomes for which data were sought. Specify whether all results that were compatible with each outcome domain in each study were sought (e.g. for all measures, time points, analyses), and if not, the methods used to decide which results to collect. | Page 7 |
|  | 10b | List and define all other variables for which data were sought (e.g. participant and intervention characteristics, funding sources). Describe any assumptions made about any missing or unclear information. | Page 7 |
| Study risk of bias assessment | 11 | Specify the methods used to assess risk of bias in the included studies, including details of the tool(s) used, how many reviewers assessed each study and whether they worked independently, and if applicable, details of automation tools used in the process. | Pages 7/8 |
| Effect measures | 12 | Specify for each outcome the effect measure(s) (e.g. risk ratio, mean difference) used in the synthesis or presentation of results. | Page 8 |
| Synthesis methods | 13a | Describe the processes used to decide which studies were eligible for each synthesis (e.g. tabulating the study intervention characteristics and comparing against the planned groups for each synthesis (item #5)). | Pages 6-8 |
|  | 13b | Describe any methods required to prepare the data for presentation or synthesis, such as handling of missing summary statistics, or data conversions. | Page 8 |
|  | 13c | Describe any methods used to tabulate or visually display results of individual studies and syntheses. | Page 7, Tables 1-3 |
|  | 13d | Describe any methods used to synthesize results and provide a rationale for the choice(s). If meta-analysis was performed, describe the model(s), method(s) to identify the presence and extent of statistical heterogeneity, and software package(s) used. | Pages 7/8 |
|  | 13e | Describe any methods used to explore possible causes of heterogeneity among study results (e.g. subgroup analysis, meta-regression). | Page 8 |
|  | 13f | Describe any sensitivity analyses conducted to assess robustness of the synthesized results. | Page 8 |
| Reporting bias assessment | 14 | Describe any methods used to assess risk of bias due to missing results in a synthesis (arising from reporting biases). | Page 7 |
| Certainty assessment | 15 | Describe any methods used to assess certainty (or confidence) in the body of evidence for an outcome. | Page 7/8 |
| **RESULTS** | | |  |
| Study selection | 16a | Describe the results of the search and selection process, from the number of records identified in the search to the number of studies included in the review, ideally using a flow diagram. | Pages 8/9, Figure 1 |
|  | 16b | Cite studies that might appear to meet the inclusion criteria, but which were excluded, and explain why they were excluded. | Pages 8/9, Figure 1, Pages 19-25 |
| Study characteristics | 17 | Cite each included study and present its characteristics. | Table 1, Pages 19-25 |
| Risk of bias in studies | 18 | Present assessments of risk of bias for each included study. | Supplemental Table S1 |
| Results of individual studies | 19 | For all outcomes, present, for each study: (a) summary statistics for each group (where appropriate) and (b) an effect estimate and its precision (e.g. confidence/credible interval), ideally using structured tables or plots. | Pages 10-13, Figure 2, Supplemental Figures |
| Results of syntheses | 20a | For each synthesis, briefly summarise the characteristics and risk of bias among contributing studies. | Pages 10-13, Figure 2, Supplemental Figures |
|  | 20b | Present results of all statistical syntheses conducted. If meta-analysis was done, present for each the summary estimate and its precision (e.g. confidence/credible interval) and measures of statistical heterogeneity. If comparing groups, describe the direction of the effect. | Pages 10-13, Figure 2, Supplemental Figures |
|  | 20c | Present results of all investigations of possible causes of heterogeneity among study results. | Pages 10-13 |
|  | 20d | Present results of all sensitivity analyses conducted to assess the robustness of the synthesized results. | Pages 10-13 |
| Reporting biases | 21 | Present assessments of risk of bias due to missing results (arising from reporting biases) for each synthesis assessed. | Pages 10-13 |
| Certainty of evidence | 22 | Present assessments of certainty (or confidence) in the body of evidence for each outcome assessed. | Pages 10-13 |
| **DISCUSSION** | | |  |
| Discussion | 23a | Provide a general interpretation of the results in the context of other evidence. | Pages 13-17 |
|  | 23b | Discuss any limitations of the evidence included in the review. | Pages 17/18 |
|  | 23c | Discuss any limitations of the review processes used. | Pages 17/18 |
|  | 23d | Discuss implications of the results for practice, policy, and future research. | Pages 16/17 |
| **OTHER INFORMATION** | | |  |
| Registration and protocol | 24a | Provide registration information for the review, including register name and registration number, or state that the review was not registered. | n/a |
|  | 24b | Indicate where the review protocol can be accessed, or state that a protocol was not prepared. | n/a |
|  | 24c | Describe and explain any amendments to information provided at registration or in the protocol. | n/a |
| Support | 25 | Describe sources of financial or non-financial support for the review, and the role of the funders or sponsors in the review. | Page 18 |
| Competing interests | 26 | Declare any competing interests of review authors. | Page 2 |
| Availability of data, code and other materials | 27 | Report which of the following are publicly available and where they can be found: template data collection forms; data extracted from included studies; data used for all analyses; analytic code; any other materials used in the review. | Page 18 |

*From:*  Page MJ, McKenzie JE, Bossuyt PM, Boutron I, Hoffmann TC, Mulrow CD, et al. The PRISMA 2020 statement: an updated guideline for reporting systematic reviews. BMJ 2021;372:n71. doi: 10.1136/bmj.n71
